# Self‐Reinforced Inductive Effect of Symmetric Bipolar Organic Molecule for High‐Performance Rechargeable Batteries

**DOI:** 10.1002/advs.202301993

**Published:** 2023-09-26

**Authors:** Giyeong Son, Vitalii Ri, Donghan Shin, YounJoon Jung, Chan Beum Park, Chunjoong Kim

**Affiliations:** ^1^ Department of Materials Science and Engineering Korea Advanced Institute of Science and Technology (KAIST) 335 Science Road Daejeon 34141 Republic of Korea; ^2^ Department of Materials Science and Engineering Chungnam National University 99 Daehak‐ro Daejeon 34134 Republic of Korea; ^3^ Department of Chemistry Seoul National University 1 Gwanak‐ro Seoul 08826 Republic of Korea

**Keywords:** all‐organic batteries, Li‐ion batteries, organic electrodes, self‐reinforced inductive effect, symmetric bipolar organic molecules

## Abstract

Herein, the self‐reinforced inductive effect derived from coexistence of both *p*‐ and *n*‐type redox‐active motifs in a single organic molecule is presented. Molecular orbital energy levels of each motif are dramatically tuned, which leads to the higher oxidation and the lower reduction potentials. The self‐reinforced inductive effect of the symmetric bipolar organic molecule, *N,N*’‐dimethylquinacridone (DMQA), is corroborated, by both experimental and theoretical methods. Furthermore, its redox mechanism and reaction pathway in the Li^+^‐battery system are scrutinized. DMQA shows excellent capacity retention at the operating voltage of 3.85 and 2.09 V (vs Li^+^/Li) when used as the cathode and anode, respectively. Successful operation of DMQA electrodes in a symmetric all‐organic battery is also demonstrated. The comprehensive insight into the energy storage capability of the symmetric bipolar organic molecule and its self‐reinforced inductive effect is provided. Thus, a new class of organic electrode materials for symmetric all‐organic batteries as well as conventional rechargeable batteries can be conceived.

## Graphical abstract

The authors scrutinize a self‐reinforced inductive effect in a bipolar molecule (*N,N* *'*‐dimethylquinacridone) through experimental and theoretical methods. This effect originates from the coexistence of *p*‐ and *n*‐type motifs, and tunes molecular orbital energy levels, leading to a wider potential gap between its oxidation and reduction. Feasible symmetric all‐organic battery based on *N,N'*‐dimethylquinacridone is also demonstrated for the first time.

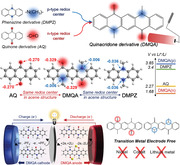

## 1 Introduction

Conventional lithium‐ion batteries utilize transition metal oxide and graphite as the cathode and anode materials, respectively.^[^ ^1^ ^]^ The state‐of‐the‐art electrode materials have been extensively investigated through past decades, however, they nearly reach the performance ceiling in the energy storage capability in terms of energy density and power. In particular, most cathode materials contain expensive and toxic elements like Co, Ni, etc., which casts doubt on the sustainable use of batteries. Recently, organic materials are considered attractive alternatives to inorganic‐based electrode materials. Organic electrode materials consist of light and inexpensive elements such as C, H, O, N, and S.^[^ ^2^ ^]^ The chemical diversity of organic compounds enables design flexibility in the molecular structure, which can be advantageous to tailor the physical and chemical properties of the materials, ultimately tuning the electrochemical properties. Ultimately, they can provide high energy density, fast electrochemical response, environmental benignity, and cost competitiveness compared with conventional inorganic‐ or metal‐based electrodes.^[^ ^2^, ^3^ ^]^ Therefore, various organic molecules with redox‐active motifs have been explored as electrode materials.^[^ ^2^ ^]^

Various efforts such as substitution, polymerization, π−conjugation, salification, biomimetics, etc. have been devoted to ameliorate the electrochemical properties of organic molecules.^[^ ^2^, ^3^, ^4^ ^]^ In particular, the introduction of electron‐withdrawing groups (EWG) (e.g., ─CF_3_, ─CN, ─F, etc.) or electron‐donating groups (EDG) (e.g., ─NH_2_, ─CH_3_,‐─OCH_3_, etc.) to the molecular structure is reported to tune the operating voltage window of the organic electrode materials.^[^ ^4^, ^5^ ^]^ However, critical issues such as low redox potential (<3 V), severe dissolution into the conventional organic electrolyte system, poor structural stability, etc., have significantly limited the practical application of single organic molecules to the current energy storage system.^[^ ^2^ ^]^ Even though several organic molecules showed higher redox voltage than 3 V (vs Li^+^/Li), poor cycling stability needs to be improved in a timely manner for their use.

Herein, we unveil that the simultaneous hybridization of the *p*‐type (─N(CH_3_)─) with the *n*‐type (C═O) redox centers in a single symmetric organic molecule comprises outstanding battery performance. It should be noted that discussed strategy thoroughly differs from the earlier studies that introduced monotonous substitution of EWG or EDG into the molecular structure,^[^ ^4^, ^5^, ^6^ ^]^ or linking of *p*‐ and *n*‐type redox centers by a redox‐inactive linker.^[^ ^7^ ^]^ In order to tune the operation voltage window more dramatically without significant loss of energy density, both EDG and EWG directly work as redox centers by themselves. Moreover, EDG and EWG are positioned in the same carbon ring of the symmetric bipolar molecule to maximize the inductive effect, thus leading to the self‐reinforced inductive effect followed by a remarkable enhancement of the energy storage capability. Few studies have been recently reported by similar strategies, where direct fusion of the two redox motifs with opposing nature was carried out.^[^ ^7^, ^8^ ^]^

We introduced quinacridone (QA) derivatives as a new class of symmetric bipolar molecules for organic electrode materials. The QA pigments have been traditionally used in industrial colorant applications like paints and inks because of their color indication and weather fastness.^[^ ^9^ ^]^ Recently, QA derivatives are also applied to organic optoelectronic devices including organic light‐emitting diodes, organic semiconductors, organic solar cells, organic field‐effect transistors, etc., owing to their high charge carrier mobility.^[^ ^10^ ^]^ The intermolecular hydrogen bond‐donating nature of the N─H group and π−π stacking interaction in QA derivatives contribute to the good photo‐, thermal, electrochemical, and structural stabilities of supramolecular organic semiconductors. Interestingly, QA derivatives show very stable and reversible redox reactions in their both *n*‐type and *p*‐type states, which is highly desired in energy storage materials.^[^ ^11^ ^]^ Although the energy storage capability of QA derivatives was reported in Li^+^‐ion battery systems, their redox mechanism was not thoroughly studied.^[^ ^12^ ^]^

**Figure 1:**
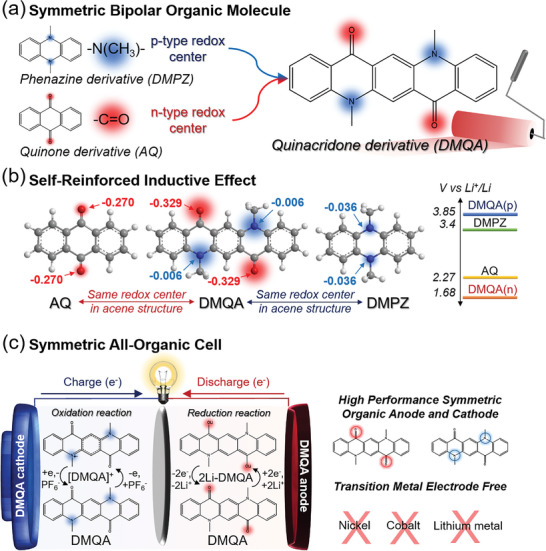
The symmetric bipolar organic molecule with self‐reinforced inductive effect for enhanced battery performance. a) DMQA was used as the model system for the symmetric bipolar organic molecule that contains two tertiary amine groups (*p*‐type motif) and two carbonyl groups (*n*‐type motif), which are known to work as redox centers of DMPZ and AQ, respectively. b) Hirshfeld charge population of AQ, DMQA, and DMPZ. DMQA enables higher charge separation between *p*‐ and *n*‐type motifs compared with those of DMPZ and AQ, resulting in a strong self‐reinforced inductive effect. c) The proposed symmetric all‐organic cell assembled with DMQA electrodes as both the cathode and anode.

In this study, we demonstrate the self‐reinforced inductive effect in the symmetric bipolar QA derivative, *N,N'*‐dimethylquinacridone (DMQA), which led to the outstanding battery performance in the lithium‐ion rechargeable battery system, as shown in **Figure** **1**. We elaborated the self‐reinforced inductive effect of the bipolar symmetric single molecule by thorough comparative studies about DMQA with the mono‐polar organic molecules, anthraquinone (AQ), and dimethylphenazine (DMPZ), that have the same *p*‐ and *n*‐type redox‐active motifs, respectively. The discharge voltage of DMQA, 3.85 V (vs Li^+^/Li), during its oxidation reaction (at the *p*‐type redox center), is one of the highest among those of single organic electrode materials available in the earlier studies, as shown in Figure 1 and Table S1 (Supporting Information). The DMQA cathode could deliver no capacity fading up to 500 cycles after the activation process of the electrode. Meanwhile, the reduction reaction of DMQA allowed an average charge voltage of 2.09 V with relatively stable capacity retention (81% at the 100th cycle). We scrutinized the redox mechanism and reaction pathway of DMQA in the lithium‐ion rechargeable battery system by both experimental and theoretical identification. In addition, we demonstrated that the DMQA molecule can be utilized as both cathode and anode materials in the symmetric all‐organic cell. Therefore, a new class of bipolar symmetric single molecules, DMQA, with high battery performance could be proposed. Our work describes a comprehensive understanding of the self‐reinforced inductive effect of bipolar symmetric organic molecules in detail, which can provide the rationalized design rule toward deliberate tailoring of physical and chemical properties of organic molecules.

## 2 Results and Discussion

### 2.1 Computational Studies of Self‐Reinforced Inductive Effect

The distribution of the charges in organic molecules is significantly affected by the introduction of EWGs or EDGs to its structure, which leads to the so‐called inductive effect.^[^ ^13^ ^]^ The inductive effect adjusts the working voltage range of organic electrode materials because the introduction of EWGs and EDGs tunes the energy level of the lowest unoccupied molecular orbital (LUMO) and highest occupied molecular orbital (HOMO) of organic molecules, respectively.^[^ ^4^, ^5^, ^6^ ^]^ From this perspective, the coexistence of carbonyl groups (*n*‐type redox centers) and tertiary amine groups (*p*‐type redox centers) in the same carbon ring, which works as EWGs and EDGs, is considered to result in the self‐reinforced inductive effect, and thus oxidation and reduction reactions of DMQA are expected to occur at the higher charging and lower discharging voltages, respectively.

**Figure 2:**
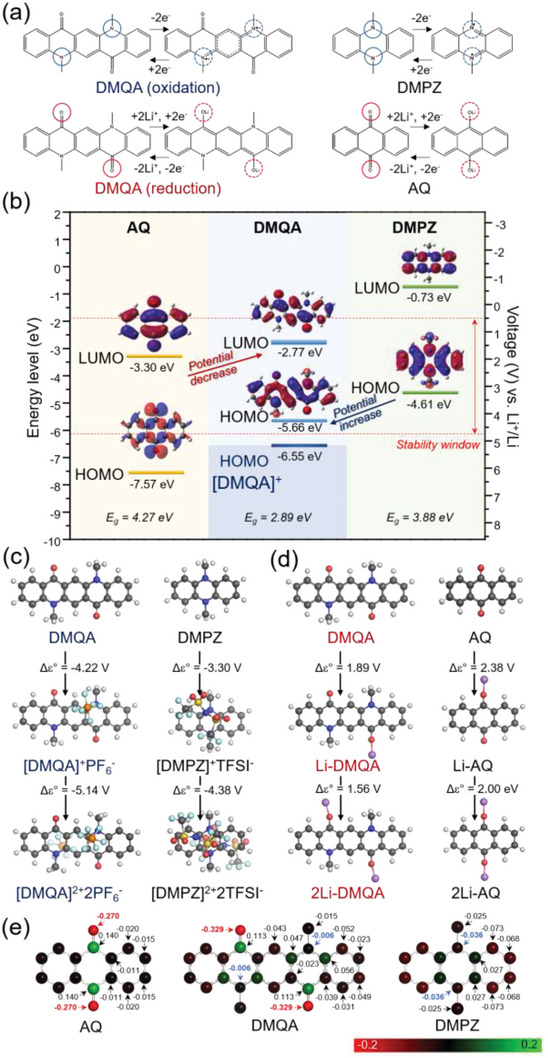
Self‐reinforced inductive effect of DMQA predicted by DFT calculation. a) Chemical structures and redox mechanisms of DMQA, AQ, and DMPZ. b) Molecular orbital structures and energy diagrams of DMQA, AQ, and DMPZ. LUMO of DMQA (−2.77 eV) is higher than that of AQ (−3.30 eV). Whereas the HOMO of DMQA (−5.66 eV) is lower than that of DMPZ (−4.61 eV). This indicates that DMQA has a lower reduction voltage than that of AQ in the reduction reaction and a higher oxidation voltage than that DMPZ in the oxidation reaction. c) Electrochemical potentials of DMQA and DMPZ during anion‐coupled oxidation. d) Electrochemical potentials of DMQA and AQ during Li^+^‐coupled reduction. e) Hirshfeld partial charges of DMQA, AQ, and DMPZ. Oxygen atoms of DMQA tend to have more electronegative charge than those of AQ. Nitrogen atoms in DMQA are more electro‐positive compared with those of DMPZ.

In order to elucidate the self‐reinforced inductive effect in the bipolar DMQA molecule, we compared DMQA with mono‐polar organic molecules, DMPZ and AQ, which have the same *p*‐ and *n*‐type redox centers with DMQA, respectively, in the acene framework. The *p*‐type redox centers of DMPZ and the *n*‐type redox centers of AQ are two tertiary amine groups and two carbonyl groups, respectively, in the anthracene structure. We carried out the density functional theory (DFT) calculation about the redox‐active motifs in DMQA, DMPZ, and AQ according to the previously proposed mechanisms, as shown in **Figure** **2**a.^[^ ^5^, ^11^, ^14^ ^]^ The energy levels of HOMOs and LUMOs of DMQA, DMPZ, and AQ were calculated to assess the redox characteristics. As depicted in Figure 2b, the energy levels of LUMOs of AQ and DMQA are −3.30 and −2.77 eV, which correspond to 1.91 and 1.38 V (vs Li^+^/Li), respectively. (Hereafter, voltage (V) is referenced by the standard redox potential of Li (V vs Li^+^/Li) unless specified.) The more positive energy of LUMO in DMQA is attributed to the less electron affinity of DMQA, therefore lowering the operating voltage compared with AQ during its reduction reaction. In contrast, the energy levels of HOMOs of DMPZ and DMQA are −4.61 and −5.66 eV, corresponding to 3.22 and 4.27 V, respectively. The more negative energy of HOMO in DMQA indicates the greater ionization energy in DMQA, which leads to a higher operating voltage than that of DMPZ during its reaction. Furthermore, the energy gap (*E*_g_) between HOMO and LUMO levels of DMQA (2.89 eV) is much lower than those of AQ (4.27 eV) and DMPZ (3.88 eV). The lower bandgap of DMQA is accounted for the facile intra‐charge transport in the molecular structure, thus possibly leading to a fast response during battery operation, as discussed later.

In addition, we directly derived cell potentials of DMQA, DMPZ, and AQ during the redox reaction (Note S1, Supporting Information, Figure 2c,d).^[^ ^15^ ^]^ Since the oxidation of DMQA or DMPZ is accompanied by the anion‐association process, PF_6_^−1^ and TFSI^−1^ were chosen as counter anions, X^−1^, to calculate cell potentials during the oxidation of DMQA and DMPZ, respectively, in order to synchronize theoretical calculation with the experimental results in the following chapter. The negligible difference in the computed cell potentials was observed regardless of the kind of counter anions, as depicted in Figure S1 (Supporting Information). The cell potentials of DMQA were calculated to be 4.22 and 5.14 V at the 1st oxidation (i.e., the 1st electron extraction) and the 2nd oxidation (i.e., the 2nd electron extraction), respectively, whereas those of DMPZ were 3.30 and 4.38 V at the 1st and the 2nd oxidation, respectively. The comparison between computed cell potentials of DMQA and DMPZ again convinces that higher energy is demanded the oxidation reaction in DMQA compared with DMPZ, which is consistent with the calculated energy levels of HOMOs and LUMOs.

Meanwhile, the reduction of DMQA and AQ occurs concurrently with cation association. Only Li^+^ was considered as the countercation that takes part in the cation association. The computed cell potentials of DMQA are calculated to be 1.89 and 1.56 V versus Li^+^/Li at the 1st and the 2nd electron insertion, respectively, which are lower than those of AQ (2.38 and 2.00 V, respectively) (Figure 2d). For further validation of the self‐reinforced inductive effect in DMQA, we designed the DMQA derivative, DMQA‐V, in which *p*‐ and *n*‐type motifs are located in different carbon rings. Comparison between cell potentials of DMQA and DMQA‐V evidently confirmed that the inductive effect of DMQA is stronger than that of DMQA‐V (see Note S2 and Figure S2, Supporting Information). Our theoretical findings indicate that the coexistence of the carbonyl group (the *n*‐type redox center) and the tertiary amine group (the *p*‐type redox center) in the same carbon ring of DMQA leads to the self‐reinforced inductive effect in DMQA.

In order to investigate charge distribution in the molecule, we calculated the Hirshfeld charge population of DMQA, DMPZ, and AQ molecules (Figure 2e). The partial charge of nitrogen atoms in the tertiary amine groups of DMQA that are the major redox contributors during oxidation reaction is higher than that in DMPZ by 0.030. In a similar manner, the partial charge of oxygen atoms in the carbonyl group of DMQA that are the major redox contributors during the reduction reaction is lower than that in AQ by 0.059. The opposite change of partial charges in nitrogen and oxygen atoms consistently corroborates the self‐reinforced inductive effect in DMQA. The coexistence of *p*‐type and *n*‐type redox motifs in a DMQA molecule affects the charge distribution, rendering the shift of redox potentials of DMQA during its oxidation and reduction reactions from those of DMPZ and AQ, respectively, as discussed earlier.

### 2.2 Electrochemical Signature of Self‐Reinforced Inductive Effect

**Figure 3:**
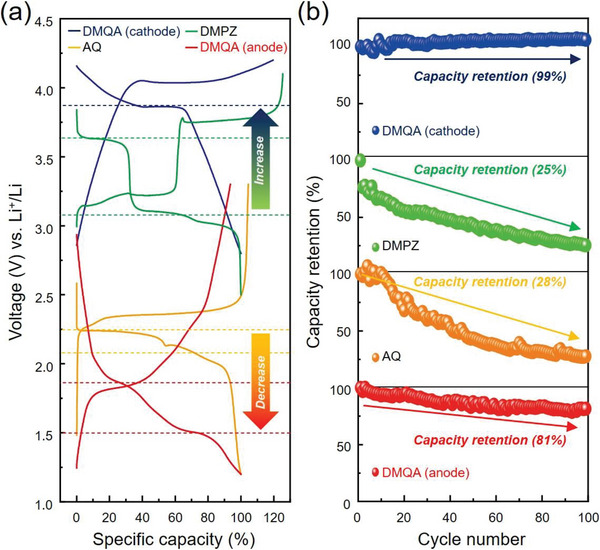
The self‐reinforced inductive effect on the electrochemical properties of DMQA. a) The voltage profiles of AQ (orange), DMPZ (green), the DMQA anode (red), and the DMQA cathode (blue) in order to investigate the difference in the electrochemical signature among the acene‐structure‐based organic single molecules. b) The capacity retentions of AQ (orange), DMPZ (green), the DMQA anode (red), and DMQA cathode (blue), emphasizing improved cycling stability of the DMQA during both oxidation and reduction reactions.

We investigated the self‐reinforced inductive effect of DMQA on electrochemical properties. We carried out a series of galvanostatic (dis)charging tests about DMQA, AQ, and DMPZ. Electrodes with DMQA, DMPZ, and AQ were prepared by simple dough preparation using the dry mixing method as described in Experimental Section in detail. Galvanostatic (dis)charging test conditions were chosen based on previously reported studies about DMPZ and AQ (see the experimental protocol in Experimental Section).^[^ ^5^, ^6^, ^14^, ^16^ ^]^ The representative voltage profiles of electrodes are presented in **Figure** **3**. A detailed study of electrochemical properties will be discussed in the following chapter. The capacity versus voltage profiles and cycle retention of DMPZ and AQ show good agreement with the previous studies.^[^ ^5^, ^6^, ^14^, ^16^ ^]^

The oxidation and reduction voltages of organic electrodes are very consistent with those derived from the HOMO and LUMO values of DMQA, DMPZ, and AQ (Figure 2b). The oxidation of DMQA occurs at a higher voltage than that of DMPZ (4.03 V vs 3.23 V) while the reduction of DMQA proceeds at a lower voltage than that of AQ (1.90 V vs 2.25 V). The electrochemical signatures strongly support the advantageous impact of the self‐reinforced inductive effect in DMQA by simultaneous integration of two electrically opposite types of functional groups: the electron‐withdrawing carboxyl group (C═O) and the electron‐donating tertiary amine group (─N(CH_3_)─). Remarkably high oxidation and low reduction voltages of DMQA are beneficial to the operation of DMQA as the positive and negative electrode, respectively, for lithium‐ion batteries. However, the second electron extraction of DMQA could not be observed albeit in its two *p*‐type redox centers. This can be attributed to the too deep HOMO level of [DMQA]^+^, −6.55 eV, corresponding to the voltage above 5 V, which is far beyond the electrochemical stable window of the electrolyte.^[^ ^17^ ^]^ Interestingly, the cycle retention of DMQA is significantly enhanced in both oxidation reaction (>99% (DMQA) versus 25% (DMPZ) after the 100th cycle) and reduction reaction (81% (DMQA) versus 28% (AQ) after the 100th cycle). Enhanced cycling stability of DMQA can be accounted for a zigzag structure (Figure S3, Supporting Information), which ameliorates the mechanical contact between DMQA and the carbon additive by strong *π−π* interaction. In addition, the steric hindrance originated from the presence of two pairs of EWGs and EDGs can suppress the undesirable side reaction with the electrolyte.^[^ ^18^ ^]^

### 2.3 Redox Activity of DMQA and Its Utilization for the Cathode and Anode

DMQA contains two tertiary amine groups (─N(CH_3_)─) and two carbonyl groups (─C═O) in its molecular structure that enable oxidation and reduction reactions, respectively.^[^ ^14^, ^19^ ^]^ The oxidation of DMQA proceeds through electron withdrawal at tertiary amine groups accompanied with the counter‐anion association. Meanwhile, reduction of DMQA occurs through electron insertion at carbonyl groups accompanied with Li^+^‐association. Since the DMQA molecule contains two *p*‐ and *n*‐type redox centers, two consecutive one‐electron transfer processes are expected to occur during each oxidation and reduction reaction of DMQA. Based on the potential regions at which redox reactions of DMQA take place, the oxidation and reduction reaction of DMQA enables DMQA to work as the cathode and anode material, respectively. The theoretical capacity corresponding to the one‐electron transfer in DMQA is ≈79 mAh g^−1^.

**Figure 4:**
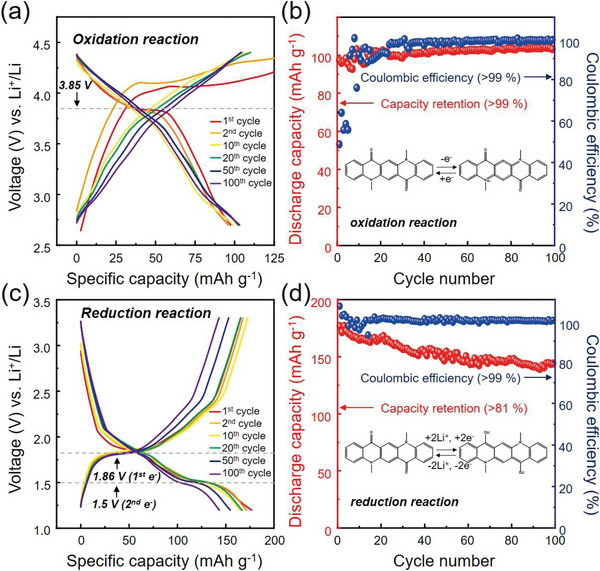
Electrochemical properties of the DMQA cathode and anode. a) Voltage profiles of the DMQA cathode cycled at the current density of 50 mA g^−1^ in the voltage range between 2.7 and 4.4 V (vs Li^+^/Li). b) Capacity retention and coulombic efficiency of the DMQA cathode under the same cycling condition. The inset of (b) shows the schematic redox reaction of the DMQA cathode. c) Voltage profiles of the DMQA anode cycled at a current density of 50 mA g^−1^ in the voltage range between 1.2 and 3.3 V (vs Li^+^/Li). d) Capacity retention and coulombic efficiency of the DMQA anode at the same conditions. The inset of (d) shows the schematic redox reaction of the DMQA anode.

The electrochemical properties of the DMQA electrode were evaluated by assembling a half‐cell structure with the Li metal as the both counter and reference electrode. The galvanostatic cycling tests were carried out at 50 mA g^−1^. A mixture of ethylene carbonate, diethyl carbonate and ethyl methyl carbonate (EC/DMC/EMC), or tetraglyme glycol dimethyl ether (TEGDME) with lithium hexafluorophosphate (LiPF_6_) or lithium bis(trifluoromethanesulfonyl)imide (LiTFSI) and concentrations (1, 2, or 3 m) were utilized as electrolytes. The oxidation reaction of DMQA was investigated in the voltage range between 2.7 and 4.4 V. The voltage window was carefully chosen considering the vulnerability of the electrolyte at high voltage. In the meantime, the reduction reaction of DMQA was studied in the voltage range between 1.2 and 3.3 V. Interestingly, the best performances of DMQA as the cathode (**Figure** **4**a,b) and the anode (Figure 4c,d) material were achieved in the different type of the electrolyte because DMQA showed strong electrolyte dependency, as discussed in the following section.

### 2.4 Charge Storage Capability of the DMQA Cathode with High Voltage and Superior Cycling Stability via Oxidation Reaction at the *p*‐Type Redox Center

First, we scrutinized the oxidation reaction of DMQA for the application of DMQA to the stable high‐voltage organic cathode material. The electrolyte screening was carried out, which revealed that the oxidation reaction of DMQA is sensitive to the solvent of the electrolyte (Figures S4–S9, Notes S3 and S4, Supporting Information). Among the various electrolyte systems, the DMQA cathodes with the carbonate‐based electrolyte showed consistent voltage profiles and discharge capacities during repeating electrochemical cycles, which is indicative of the stable operation of the cathode. The most stable battery performance with the initial discharge capacity of 97 mAh g^−1^ was achieved in the DMQA cathode using the 1 m LiPF_6_ in EC/DMC/EMC electrolyte, which clearly showed the distinct plateau at ≈3.85 V (Figure 4a; Table S2, Supporting Information). This voltage value is one of the highest values among organic cathodes based on single organic molecules available from the literature, as tabulated in Table S1 (Supporting Information). Therefore, the 1 m LiPF_6_ in EC/DMC/EMC electrolyte was utilized for the further electrochemical analysis and redox mechanism study of the DMQA cathode. The key performances of the DMQA cathode are presented in Table S2 (Supporting Information). Since the voltage for the second electron extraction is located far beyond the stable electrochemical window of the electrolyte as aforementioned,^[^ ^17^ ^]^ only one electron reaction at the *p*‐type redox center reversibly proceeds during the (dis) charging reaction of the DMQA cathode.

Despite low coulombic efficiency (C.E.) in the initial cycle, C.E. was gradually increased up to higher than 99% as the following cycles proceeded, and the DMQA cathode exhibited outstanding electrochemical stability without any noticeable capacity loss. The discharge capacity of 104 mAh g^−1^ was achieved at the 100th cycle, as shown in Figure 4b, and was well preserved even up to the 500th cycle (Figure S10, Supporting Information). Considering the capacity contribution of the carbon additive at the given voltage range (Figures S11 and S12, Supporting Information), the actual capacity of the DMQA cathode is ≈60 mAh g^−1^, which is ≈76% of the theoretical capacity assuming the one‐electron transfer of the DMQA cathode. Low C.E. in the initial cycles could be attributed to several reasons: 1) electrolyte decomposition at the lithium anode and formation of solid‐electrolyte interphase (SEI); 2) electrolyte decomposition at the DMQA cathode and formation of the cathode‐electrolyte interphase (CEI) at the high voltage (>4.2 V); 3) dissolution of [DMQA]^+^ followed by shuttle effect and irreversible reaction on the surface of Li; 4) electropolymerization of DMQA or [DMQA]^+^ at high voltage.^[^ ^20^ ^]^ Additional experiments were performed in order to address each of the above‐mentioned reasons, however, it is complicated to distinguish their contribution and degree.

First, a significant change in the cycled lithium anodes was observed as evidenced by photographs of cells disassembled after 1 and 10 cycles (Figure S13, Supporting Information). In order to investigate its origins, scanning electron microscopy (SEM) and X‐ray photoelectron spectroscopy (XPS) characterization of the lithium anode was carried out (Figures S14–S18, Supporting Information). The surface of the fresh lithium foil looks uniform and smooth from the top‐view observation (Figure S14a, Supporting Information). The SEM image from the cross‐sectional view also revealed the relatively smooth surface of the fresh Li foil (Figure S14b, Supporting Information). However, even after the 1st cycle, the lithium surface revealed significant morphological changes including loss of smoothness, formation of voids, etc., due to the irreversible Li plating and stripping, as shown in Figure S15a (Supporting Information). The top‐view image at the higher magnification, Figure S15b (Supporting Information), visualized small needle‐like shaped particles on the surface as denoted by arrows. Based on their morphology, lithium dendrites are considered to form. Such morphological change could not be clearly observed in the cross‐sectional images (Figures S15c, Supporting Information). After 20 repeated cycles, an extensive plating/stripping process led to severe morphological changes on the surface of lithium metal (Figure S16, Supporting Information), which mainly resulted from the formation of the SEI layer, Li dendrite, dead Li, and so on. The surface of Li metal film did not show film‐like morphology anymore, as presented in Figure S16a (Supporting Information). In the SEM image at the higher magnification, Figure S16b (Supporting Information), only spherical‐shaped morphologies were observed. In contrast to the cycled Li anode for only 1 cycle, the SEI layer is clearly visible and its thickness is estimated to be several‐micrometer‐thick as can be seen in the peeled layer from the cross‐sectional view (Figure S16c, Supporting Information). Interestingly, sharp needle‐like particles could not be observed possibly owing to the suppression of the Li dendrite by a stable SEI layer.

For the complemental study, XPS characterization of the lithium anodes was performed in order to chase the compositional change on the Li surface during battery operation (see Figures S17 and S18, Supporting Information). Even before the electrochemical cycling, the composition of the fresh lithium anode surface was quite complicated and composed of various Li compounds such as Li_2_O, LiOH, Li_2_CO_3_, LiF, etc. The presence of a significant amount of chemical species as well as the absence of Li metal is attributed to the unavoidable reaction with moisture, air, etc., even in the glovebox (H_2_O and O_2_ < 0.1 ppm) (Figure S17, Supporting Information). After the initial cycle, significant changes in the composition of the Li surface were detected. First, more carbonaceous compounds could be found on the surface in the form of C─C, C─H, and C─O in C 1s spectra. Such a high increase in carbon content is indicative of the formation of the organic‐based SEI layer. Meanwhile, the peak at 51.1 eV in Li 1s spectra could be attributed to Li metal (Li^0^), which resulted from the exposed lithium surface or newly formed dendrites. Of particular note is that nitrogen could be probed on the cycled Li metal surface, which did not exist in the fresh Li foil. Considering the absence of nitrogen in the electrolyte composition, the emergence of the new peak in N 1s is ascribed to the nitrogen from the shuttled DMQA. N 1s spectra indicated that most nitrogen species exist in the pyridinic form. After 20 cycles, the notable changes are the increase in C─O species as shown in O 1s and C 1s spectra accompanied with an increase of nitrogen content on the surface compared with the Li anode that was cycled for only one cycle (see the O 1s, C 1s, and N 1s spectra in Figure S17, Supporting Information, and the composition change in Figure S18, Supporting Information). The change in the composition of the Li anode suggests further growth of the organic‐based SEI layer and the continuous contribution of DMQA species to the process of the SEI layer. Moreover, the disappearance of Li^0^ metal peak suggests the absence of exposed lithium metal or dendrites by further growth of the SEI layer, which correlates well with SEM images.

We believe our findings about the Li anode provide convincing evidence about (1) the formation of the SEI layer on the lithium anode and (2) the shuttle of DMQA species to the anode side and its participation in the SEI formation process. The detailed deconvolution of XPS spectra was not performed due to the complexity of the spectra. However, peak positions are tabulated in Table S3 (Supporting Information).

The shuttle effect resulted from the dissolution of DMQA, formation of CEI, and possible polymerization of DMQA was also confirmed from the supplemental experiments (Figures S19–S21 and Note S5, Supporting Information). The shuttle effect was confirmed by the electrochemical tests performed with various cut‐off voltages. The anodic peak above 4.2 V was attributed to CEI formation and possible electropolymerization of DMQA since both could suppress the dissolution of DMQA into electrolyte as evidenced from photographs of the electrolyte solvent solution. After keeping the DMQA electrodes that were cycled for 100 cycles in the electrolyte solvent, any notable color change could not be observed as shown in Figure S22 (Supporting Information). Moreover, capacity versus voltage profiles that are gradually changed to a sloppy shape also suggest possible electropolymerization. Hence, electropolymerization is one of the possible origins of the electrochemical stabilization of DMQA.

In order to further assess the effect of SEI and CEI on the electrochemical stability of DMQA electrodes, an electrochemical test after re‐injection of the electrolyte to the cycled cells was performed (see Experimental Section and Figures S23–S25, Supporting Information). It should be noted that discharge capacity loss of the cells was not observed in the re‐assembled cells with new electrolytes, which suggests minimal loss of active materials during the reassembly of the cells. Only C.E.s of the first following cycle significantly differed among the re‐assembled cells. The cell that was assembled with the new electrolyte after the initial cycle revealed ≈40% of C.E. for the 1st following cycle (2nd cycle in Figure S23a,b, Supporting Information), which is lower than that of the reference cell (the cell without reassembly in Figure S24, Supporting Information, and in our manuscript). Moreover, the replacement of the lithium anode with the fresh Li anode did not affect CE. This confirms that electrochemical cycling for only one cycle could not lead to the formation of stable CEI and SEI layers. However, when the same test was performed for the cells that were assembled with the cycled DMQA cathode for 20 cycles and new electrolyte, the anode replacement significantly affected the C.E. of the 1st following cycle. While the use of the fresh Li anode led to an initial C.E. of 67.6%, the cell with the cycled Li anode showed an initial C.E. of 83.2% (21st and 22nd cycles in Figure S23c,d, Supporting Information, respectively). As shown in Figure S25 (Supporting Information), the re‐assembled cells showed ≈100% C.E. afterward during extended cycles. Our observation confirmed the formation of both stable SEI and CEI after at least 10 cycles as confirmed by Figures S24 and S25 (Supporting Information). In the meantime, it should be noted that the stability of the DMQA cathode could be also originated from DMQA electropolymerization at the high voltage operation, which could lead to a similar effect to the stable CEI layer. However, at the current state, it is complicated to differentiate them, so we refer to both of them as possible origins of irreversible reaction at high voltage, resulting in enhanced DMQA cathode performance.

The rate capability of the DMQA cathode was also investigated (Figure S26, Supporting Information). As a cathode, DMQA delivers 60% discharge capacity even at the high current density of 1200 mA g^−1^ (≈15 C considering the one electron transfer process) compared with that at 50 mA g^−1^ (≈0.6 C). DMQA can reveal such high rate capability during its oxidation reaction owing to the high electron mobility and intimate contact between its conjugated acene structure and the electronic additive (carbon black). In addition, the integration of electron‐donating (*p*‐type) and electron‐withdrawing (*n*‐type) redox centers in the single molecule also can contribute to the higher electronic conductivity via lowered HOMO‐LUMO gap.^[^ ^21^ ^]^ A similar effect, which results from the introduction of two different types of functional groups, was oftentimes observed in organic charge transfer complexes. When *n*‐ and *p*‐type compounds are bound to each other, layered structures are formed with ordered stacking via *π−π* interaction, thereby enabling facile charge transfer.^[^ ^22^ ^]^

### 2.5 Charge Storage Capability of the DMQA Anode at Low Voltage via Reduction Reaction at the *n*‐Type Redox Centers

The battery performance of DMQA as an anode material was investigated in the voltage range between 1.2 and 3.3 V. The DMQA electrode showed the best performance with the 3 m LiTFSI in TEGDME electrolyte, of which voltage profile and cycle retention are presented in Figure 4c,d, respectively. The results of galvanostatic (dis)charging tests for other electrolyte solutions are displayed in Figures S27–S30, Table S4, and Note S6 (Supporting Information).

The lower charging voltage of the DMQA anode is advantageous to its full cell operation. The lowest average charging voltage of ≈2.1 V (distinct two plateaus at ≈1.8 and ≈2.2 V) was observed only in the DMQA anodes with the electrolyte system using the TEGDME solvent regardless of the salt type and concentration. On the contrary, the use of the carbonate‐based electrolyte resulted in a complicated charging curve shape at a higher voltage of ≈2.5 V. The difference in the operating voltages of the DMQA anode can be ascribed to the different (de)solvation energy of lithium‐ion prior to its interaction with DMQA.^[^ ^23^ ^]^ The battery performances of the DMQA anodes under the various electrolyte systems are summarized in Table S4 (Supporting Information). Considering the best cycling stability and low overpotentials during repeating (dis)charge of the DMQA anode, 3 m LiTFSI in TEGDME electrolyte was selected for the further assessment of electrochemical properties, ex‐situ and computational study on the redox mechanism of the DMQA anode.

The DMQA anode showed a stable voltage profile even after the 100th cycle while delivering 81% of the initial discharge capacity. The C.E. was stable during repeating cycles, which showed higher than ≈99% after the initial few cycles. The relatively stable cycling performance suggests limited dissolution of DMQA as revealed from the photographs of disassembled DMQA anodes (Figure S31, Supporting Information). Considering the capacity contribution from the carbon additive (see Figure S32, Supporting Information), the DMQA anode delivered ≈133 mAh g^−1^, indicating reversible two‐electron process proceeds at the *n*‐type redox center. Two main cathodic peaks at ≈1.5 and ≈1.86 V with small shoulders at slightly higher voltages than those of main peaks (≈1.63 and ≈1.98 V) and three anodic peaks at 1.82, 2.03, and 2.31 V are clearly resolved in the dQ/dV plot (Figure S33, Supporting Information). The cathodic peak at 1.86 V with the shoulder at 1.98 V and two anodic peaks at 2.03 and 2.31 V are assigned to the redox couple of the first carbonyl motif, DMQA + e = [DMQA]^−1^. Meanwhile, the cathodic peak at ≈1.5 V with its shoulder at 1.63 V and the anodic peak at 1.82 V are corresponding to the redox couple of the second carbonyl motif, [DMQA]^−1^ + e = [DMQA]^−2^. The DMQA anode reveals a lower rate capability compared with the DMQA cathode (Figure S26, Supporting Information). Cycling at 400 mA g^−1^ (≈2.5 C) showed substantial deterioration of the capacity, which is ≈43% of the capacity achieved at 50 mA g^−1^ (≈0.3 C). Limited response in the reduction reaction of DMQA can be originated from the high viscosity of the electrolyte with high salt concentration (3 m LiTFSI in TEGDME), which retards fast Li‐ion transfer at the accelerated rate.

### 2.6 Demonstration of Successful Operation of All‐Organic‐Symmetric Cell Using DMQA as the Both Cathode and Anode Materials

**Figure 5:**
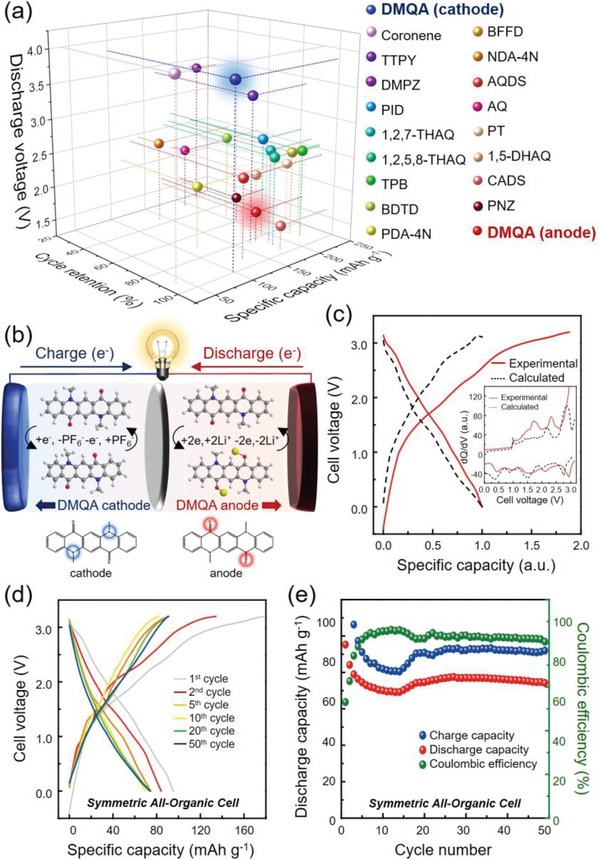
The all‐organic cell assembled with DMQA as both the anode and cathode. a) Comparison of DMQA with the previously reported organic single molecules. b) Schematic illustration of the all‐organic cell assembled with the DMQA electrodes as both the anode and cathode. During the charging process, the DMQA cathode undergoes oxidation accompanied with PF_6_^−^ anion association nearby ‐N(CH_3_)‐, while the DMQA anode undergoes reduction accompanied by Li‐ion association nearby carbonyl groups. c) Comparison of the voltage profile of all‐organic cells in the 1st cycle and calculated voltage profile. The all‐organic cell was cycled at a current density of 50 mA g^−1^ under the voltage range between 0 and 3.1 V. d) Voltage profiles of the all‐organic cell obtained at various cycles. e) Cycling stability of the all‐organic cell assembled with DMQA electrodes.

The promising battery performance of DMQA among those of other organic single molecules (**Figure** **5**a) and the particularly wide voltage gap of ≈1.8 V between reduction and oxidation reactions inspired the all‐organic‐symmetric cell that utilizes DMQA as the both anode and cathode material (Figure 5b). The galvanostatic cycling of the all‐organic‐symmetric cell was performed at the current density of 50 mAh g^−1^ in the voltage range between 0 and 3.2 V (voltage of the full cell), as presented in Figures 5c–e. For the fabrication of the symmetric cell, the cathode was stabilized for 20 cycles (see Experimental Section) prior to being assembled into the symmetric cell. The anode was directly used as prepared. Though the DMQA cathode and anode showed the best battery performance under the different electrolyte systems, the 1 m LiPF_6_ in EC/DMC/EMC electrolyte was solely utilized for the all‐organic‐symmetric full cell. The symmetric cell exhibited 95 mAh g^−1^ (normalized by the weight of DMQA in the cathode) with an average discharge voltage of 1.60 V at the 1st cycle. The capacity of the full cell coincides with that of the DMQA cathode.

The voltage profile at the 1st cycle is similar to the calculated voltage profile that is derived from the profiles of the DMQA cathode and DMAQA anode (Figure 5c). The dQ/dV curve for the 1st cycle shows the complicated shape that is a sum of electrochemical signatures of the DMQA anode and cathode (Figure 5c (inset)). The experimentally derived dQ/dV curve is well correlated with the calculated curve, showing good agreement in the positions of each peak. Therefore, the successful operation of all organic symmetric cells using DMQA as the cathode and anode could be demonstrated. However, the symmetric cell delivered poor cycling stability compared with cathode and anode half cells due to the incompatibility of the electrolyte with the DMQA anode. Detailed discussion about the degradation of the anode can be found in Figures S34, S35, and Note S7 (Supporting Information). The discharge capacities at the 50th and 100th cycles were 77% and 48% of the initial capacity, respectively (Figure 5d,e). The battery performance of the all‐organic‐symmetric cell is currently being optimized to achieve stable battery operation. Nonetheless, the successful demonstration of all‐organic batteries using DMQA as both cathode and anode presents feasibility for Li‐free and symmetric all‐organic batteries, which paves the new way for the challenge to the conventional battery and its manufacturing system.

### 2.7 Redox Mechanism of Charge Storage of DMQA at *p*‐Type and *n*‐Type Redox Centers

**Figure 6:**
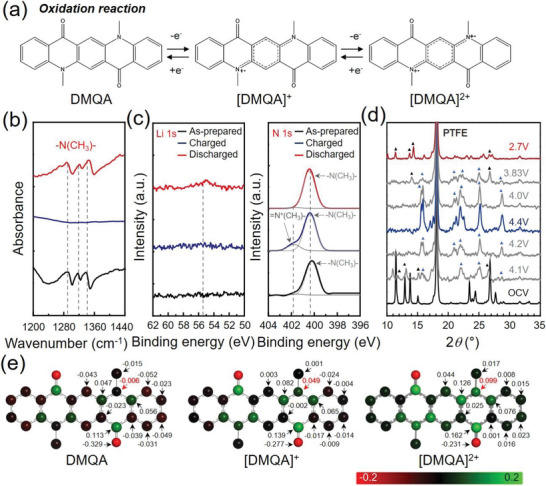
The redox mechanism of oxidation reaction of DMQA. a) The redox pathway of DMQA oxidation reaction. b) Ex‐situ FTIR spectra of ─N(CH_3_)─ obtained at the different (dis)charged states. c) XPS spectra of Li 1*s* and N 1*s* at the as‐prepared, charged, and discharged states. d) XRD patterns at the different (dis)charged states. e) Change of partial charge in DMQA during oxidation calculated by DFT methods.

The redox mechanism of QA has been studied by electrochemical and spectroscopic analysis for certain applications such as the organic semiconductor.^[^ ^11^ ^]^ In an anhydrous electrolyte solution, the first oxidation of QA derivatives drives the formation of radical cations and then the second diradical cations are formed by the second oxidation. Positive charges are localized in the vicinity of nitrogen atoms. We hypothesize that the oxidation of DMQA occurs via anion association at the nitrogen atoms in the pentacene structure during battery operation (**Figure** **6**a). We investigated the proposed reaction pathway for the oxidation reaction of DMQA at its *p*‐type redox centers by combinatorial studies based on DFT calculation and experimental analysis using Fourier transform infrared spectroscopy (FTIR) and high‐resolution X‐ray photoelectron spectroscopy (XPS). During the charge and discharge of the DMQA cathode, FTIR spectra exhibit reversible emergence and extinction of the peaks at 1280–1340 cm^−1^, which are corresponding to the vibrational modes of C─N─C bonds.^[^ ^24^ ^]^ Our observation ensures that nitrogen atoms in conjugated carbon rings are mainly responsible for the oxidation of DMQA (Figure 6b).

In the meantime, XPS analysis convinced the oxidation of DMQA at nitrogen atoms without Li^+^ insertion (Figure 6c). The Li 1*s* spectra of DMQA did not show the peak at the as‐prepared state and the (dis)charged state. In contrast, the N 1*s* spectrum of the as‐prepared DMQA electrode revealed the single peak at 400.37 eV, which originated from ─N(CH_3_)─. The N 1*s* spectrum of the 4.4 V charged DMQA cathode showed two peaks at 400.37 and 401.78 eV, which are assigned to ─N(CH_3_)─ and ═N^+^(CH_3_)─, respectively. Presence of the two bonds in the N 1*s* spectrum at the charged state results from the partial oxidation of DMQA, which correlates well with the one‐electron reaction during the oxidation of the DMQA cathode. The peak associated with ═N^+^(CH_3_)─ disappears in discharged DMQA cathode. The N 1*s* spectrum is fitted with the single peak at 400.49 eV, indicating a reversible redox reaction between DMQA and [DMQA]^+^.

In addition, X‐ray diffraction analysis (XRD) was performed in order to chase structural changes during the operation of the DMQA cathode. It should be noted that the significant decrease in crystallinity as well as the change of relative intensities among the most prominent peaks were detected in the prepared electrode compared with the DMQA powder (Figure S36, Supporting Information). The great change in XRD patterns was attributed to the grinding process of the DMQA powder with the PTFE binder and carbon additive powder. The sheer stress during the mechanical grinding could lead to the cleavage of the DMQA crystals at the planes with the weakest interplanar interaction.^[^ ^25^ ^]^ Moreover, it results in the partial amorphization of DMQA and the distribution of small stacks of DMQA molecules on the surface of carbon particles via *π−π* interactions.

The structural changes were identified during the 1st cycle of the DMQA cathode (Figure 6d). At the ≈4.1 V charged state, which corresponds to the ≈0.5 electrons withdrawn from the DMQA molecule (see Figure S37, Supporting Information), the presence of two phases was detected: 1) the DMQA initial phase with severe perturbations (black triangles) and 2) the new unidentified phases (blue triangles). The perturbations from the initial phase of DMQA could be explained by the PF_6_^−^ counter‐anion association during the charging process. The insertion of PF_6_^−^ with the relatively large size during oxidation of DMQA cathode led to further cracking at the planes of stacked DMQA. Upon further charging up to 4.4 V, the complete disappearance of the initial DMQA phase and more prominent evolution of the new phases were observed. Therefore, the new phase that appeared during the charging process can be identified by the [DMQA]^+^PF_6_^−^ phase. The recovery of the peaks albeit with a much weaker intensity that resembles the initial DMQA phase was observed during discharging process of the DMQA cathode. Our observation indicates that the phase transition between DMQA and [DMQA]^+^PF_6_^−^ is reversible despite being accompanied with a severe loss in the crystallinity during repeating cycles (Figure S38, Supporting Information). The more detailed analysis could be addressed using small‐angle X‐ray scattering (SAXS) for future work.

The redox pathway of the oxidation reaction of DMQA at *p*‐type redox center is further supported by DFT calculation. According to the Hirshfeld charge analysis, the most significant change of partial charge in DMQA occurs at nitrogen atoms and the middle carbon ring, suggesting their contribution to the oxidation reaction of DMQA (Figure 6e). In the meantime, bond length alteration analysis (see Figure S39, Supporting Information) reveals a conformational change of DMQA during oxidation. Upon the first oxidation process of DMQA, the most significant alteration in the bonding length is the shortening of C3─N5 (right side) and C3’─N5’ (left side) bonds to a similar degree. Simultaneously, the shortening of the C1─C2 bond and elongation of C2─C3 and C3─C4 bonds on both sides of DMQA occur in the middle ring, which compromises the shrinkage of the benzene ring in the middle of DMQA. Further oxidation of DMQA leads to increased alterations of the same bonds. Therefore, the electron withdrawal process results in a symmetric change in the DMQA molecule due to the effective charge distribution among the two amine groups and the conjugated structure between them (Figure 6e). The two amine groups act as major redox centers and the conjugated carbon ring between them plays a supportive role in charge distribution, preventing highly localized charge distribution followed by severe structural deformation in the DMQA molecule. Our observation about the conformational change of DMQA is consistent with that of QA.^[^ ^11^ ^]^

**Figure 7:**
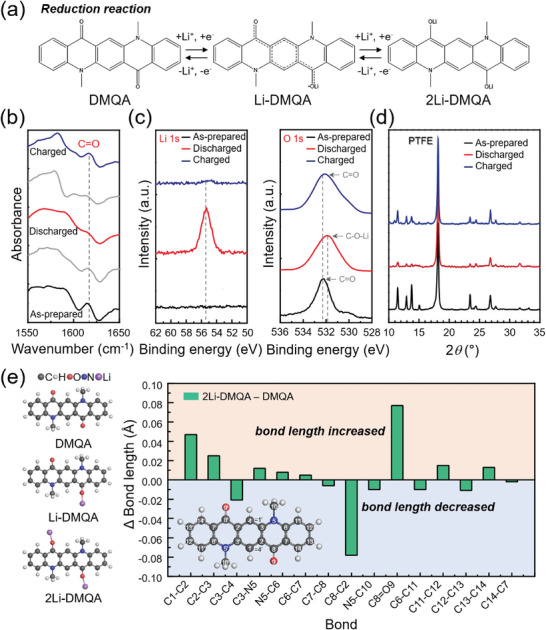
The redox mechanism of reduction reaction of DMQA. a) Li^+^‐coupled reduction reaction of DMQA. b) Ex‐situ FTIR spectra of ‐N(CH_3_)‐ at the different (dis)charged states. c) XPS spectra of Li 1*s* and O 1*s* at as‐prepared, discharged, and recharged states. d) XRD patterns harvested at as‐prepared, discharged, and recharged states. e) Molecular structures of DMQA, Li‐DMQA, and 2Li‐DMQA along with the bond length alteration of DMQA at the end of the discharge process.

In the reduction process, negative charges are reported to be localized at the oxygen atoms of QA derivatives.^[^ ^11^ ^]^ We assumed that the electron transfer reaction occurs at carbonyl groups of DMQA, as depicted in **Figure** **7**a. According to the FTIR analysis, the vibrational mode of the C═O bond at 1617 cm^−1^ disappeared at the fully discharged state. Then it was recovered after recharging, indicating that the carbonyl groups are the reversible and major *n*‐type redox centers of DMQA during the reduction reaction (Figure 7b). Meanwhile, high‐resolution XPS spectra of the DMQA anode (Figure 7c) confirmed that the reduction of DMQA is accompanied by lithium‐ion insertion and extraction. Li 1*s* spectra of the fully discharged DMQA anode showed the emergence of a peak at 55.5 eV, which can be elucidated by the insertion of Li^+^ during the reduction of DMQA to [DMQA]^2−^. The Li 1*s* peak disappeared after recharging of [DMQA]^2−^ to DMQA followed by extraction of Li^+^. The XPS spectra of O 1*s* also exhibited the commensurate peak shift of the peak at 532.2 eV. Shift of the peak to the lower energy by 0.4 eV upon discharging of the DMQA anode was clearly observed. The origin of energy shift is understood by the increase of electron density around oxygen atoms during discharging, which is consistent with the formation of C─O─Li bond as confirmed by FTIR and XPS spectra of Li 1*s*. The post‐mortem analysis using *ex‐situ* FTIR and XPS analyses showed the reversible DMQA reduction to [DMQA]^2−^ at the carbonyl groups accompanied with Li^+^ insertion during the reduction reaction of DMQA.

Ex‐situ X‐ray diffraction analysis (Figure 7d) revealed a decrease in the peak intensities of the initial DMQA phase upon reduction. However, the peak intensities were recovered by following the charging process of the DMQA anode. Compared with the huge change of XRD patterns in the DMQA cathode during its redox reaction, the DMQA anode suffers mild structural change. It can be attributed to the association of Li^+^ with small size during the redox reaction of the DMQA anode. The structural perturbation of DMQA molecules by Li^+^ is negligible.

We examined changes in bond lengths during the reduction of DMQA (Figure 7e; Figure S40, Supporting Information). The major alterations in the molecular structure of DMQA during the reduction reaction suggest a similar charge storage mechanism to that during the oxidation process. Two carbonyl groups work as main redox centers and the conjugated benzene ring in the middle of the molecular structure plays a pivotal role during the reduction reaction of DMQA. When the first electron insertion proceeds during the reduction process of DMQA, the most significant changes occur in the moiety of carbonyl groups. The elongation of the C8═O9 bond and shortening of the C8─C2 bond occurs. The analogous changes occur around another carbonyl group albeit with a less degree. The much larger change in the bond lengths (C8═O9 (1.281 Å) and C2═C8 (1.428 Å)) compared with other bonds suggests the formation of the radical anion structure, [DMQA]^•−^, as depicted in Figure 7a (middle), where negative charge is localized on oxygen atom as an unpaired electron upon reduction. In addition, the decrease in the double bond character of C8═O9 in the carbonyl group leads to higher electron delocalization in the middle benzene ring and another carbonyl group. This broad resonance structure results in the formation of the stable radical anion, [DMQA]^•−^, with Li^+^. The coordinate bond between the carbonyl oxygen and Li‐ion, O9─Li, can form. Hence, the carbonyl group that is associated with Li^+^ acts as the main redox center since the most dominant changes occur at its proximity. Meanwhile, another carbonyl group along with the conjugated center carbon ring plays a supportive role during the one‐electron reduction process.

The further reduction of DMQA leads to the formation of the second Li‐ion coordinated carbonyl group through a similar mechanism to that during the first electron insertion. It should be noted that the delocalized structure can be achieved by another lone pair on the carbonyl oxygen atom that is not associated with Li^+^, leading to the loss of radical character. This mechanism is supported by the bond lengths of C2═C8 (1.400 Å), C8═O9 (1.306 Å), and C1═C2 (1.442 Å) after the second electron insertion by further reduction, which showed a relatively large difference compared with other bonds.

Thus, the resultant symmetrical structure of 2Li‐DMQA leads to the formation of DMQA dianion coupled with two lithium ions (Figure 7a (right)), where negative charges are localized on oxygen atoms. All in all, the changes in bond lengths strongly support the molecular structures presented in Figure 7a and are well correlated with previously reported spectroelectrochemical observation during the reduction of quinacridone.^[^ ^11^ ^]^ In contrast to the conformational change during oxidation, the alterations in the bond lengths occur asymmetrically (i.e., major changes occur only around the reduced carbonyl group during the one electron insertion), which suggests an independent reduction of each carbonyl group upon two‐step reduction process. These results elaborate detailed redox pathways of charge storage in DMQA during both oxidation and reduction in the lithium‐ion battery system, as the cathode and anode, respectively. Thus, a combination of experimental analysis and theoretical calculation successfully confirmed the proposed pathways. In addition, the significant contribution of the conjugated carbon rings in the DMQA molecule to the stabilization of radical ion structures was identified, which is more prominent during the oxidation of DMQA.

## 3 Conclusion

In conclusion, we unravel that the self‐reinforced inductive effect of a symmetric bipolar single molecule can elicit a change of HOMO and LUMO energy levels, therefore significantly tuning the redox potentials. We designed the new organic electrode, DMQA, as a model system, in which a self‐reinforced inductive effect occurs in the symmetric bipolar molecular structure. We scrutinized the modulation of redox potentials in DMQA by theoretical calculation and study about the electrochemical signature. By the comparative study with the control group (DMPZ and AQ), we corroborated the self‐reinforced inductive effect in DMQA. DMQA revealed excellent battery performance as both cathode and anode materials. Hence, we, for the first time, demonstrated the operation of the all‐organic symmetric battery using DMQA molecules. We also elaborated the redox mechanism that is responsible to the charge storage of DMQA during battery operation through complemental analysis using *ex‐situ* characterizations and DFT calculation. We believe our study provides a comprehensive understanding of the self‐reinforced inductive effect of single organic molecules, which can dramatically ameliorate the electrochemical properties of organic batteries. Furthermore, we suggest the rationalized design rule toward deliberate tailoring of physical and chemical properties of organic molecules for various applications.

## Conflict of Interest

The authors declare no conflict of interest.

## Author Contributions

G.S. and V.R. contributed equally to this work. G.S. and V.R. conceived the conceptual idea. G.S. and V.R. designed all the experiments, analyzed the results, and prepared the manuscript. D.S. and Y.J. conducted theoretical calculations based on density functional theory. C.B.P. and C.K. supervised and guided the entire project.

## Supporting information

Supporting Information

## Data Availability

The data that support the findings of this study are available from the corresponding author upon reasonable request.
