# Supplementary material for: Self‐Reinforced Inductive Effect of Symmetric Bipolar Organic Molecule for High‐Performance Rechargeable Batteries
Source: Adv Sci (Weinh). 2023 Sep 26;10(31):2301993. doi: 10.1002/advs.202301993 (PMC10625108; doi:10.1002/advs.202301993)
Supplement: Supplementary file 1 — Supporting Information [file ADVS-10-2301993-s001.pdf]

ADVANCED SCIENCE
Open Access

Supporting Information

for *Adv. Sci.*, DOI 10.1002/advs.202301993

Self-Reinforced Inductive Effect of Symmetric Bipolar Organic Molecule for High-Performance Rechargeable Batteries

Giyeong Son, Vitalii Ri, Donghan Shin, YounJoon Jung, Chan Beum Park and Chunjoong Kim**

Supplementary information

Self-reinforced inductive effect of symmetric bipolar organic molecule for high performance rechargeable batteries

Giyeong Son†[a], Vitalii Ri†[b], Donghan Shin[c], YounJoon Jung[c], Chan Beum Park*[a] and Chunjoong Kim*[b]

[a] G. Son, C. B. Park
Department of Materials Science and Engineering
Korea Advanced Institute of Science and Technology (KAIST)
335 Science Road, Daejeon, 34141, Republic of Korea
E-mail: parkcb@kaist.ac.kr

[b] V. Ri, C. Kim
Department of Materials Science and Engineering
Chungnam National University
99 Daehak-ro, Daejeon, 34134, Republic of Korea
E-mail: ckim0218@cnu.ac.kr

[c] D. Shin, Y. J. Jong
Department of Chemistry
Seoul National University
1 Gwanak-ro, Seoul, 08826, Republic of Korea

† These authors contributed equally
* Corresponding authors

Abstract: Herein, we present the self-reinforced inductive effect derived from coexistence of both p- and n-type redox active motifs in a single organic molecule. Molecular orbital energy levels of each motif were dramatically tuned, which leads to the higher oxidation and the lower reduction potentials. We corroborated self-reinforced inductive effect of the symmetric bipolar organic molecule, N,N'-Dimethylquinacridone (DMQA), by both experimental and theoretical methods. Furthermore, we scrutinized its redox mechanism and reaction pathway in the Li^+-battery system. DMQA showed excellent capacity retention at the operating voltage of 3.85 V and 2.09 V (vs. Li^+/Li) when used as the cathode and anode, respectively. Successful operation of DMQA electrodes in symmetric all-organic battery was also demonstrated. We provided the comprehensive insight about energy storage capability of the symmetric bipolar organic molecule and its self-reinforced inductive effect. Thus, we could conceive the new class of organic electrode materials for symmetric all-organic batteries as well as conventional rechargeable batteries.

Table of Contents

1. Experimental section 3
2. Summary of the key battery performances of organic electrode materials 6
3. DFT calculations on inductive effect of DMQA 8
4. Additional tests on electrochemistry of DMQA 11
5. Symmetric cell with DMQA as cathode and anode 32
6. Redox mechanism of DMQA during anion- and lithium- coupled reactions 33
7. References 36

1. Experimental section

4.1. Electrode preparation

Anthraquinone (AQ) was purchased from Sigma Aldrich (USA). N,N'-Dimethylphenazine (DMPZ) and N,N'-Dimethylquinacridone (DMQA) were purchased from TCI Chemicals (Japan). AQ, DMPZ, and DMQA were utilized without further purification. DMQA and AQ electrodes were fabricated by mixing the active material, conductive carbon black (Ketjen Black, EC-600JD, LION Specialty Chemicals Co., LTD.) and binder (PTFE beads (poly(tetrafluoroethylene)), Sigma Aldrich, Japan) with the weight ratio of 40 : 40 : 20 (wt.%). Firstly, the paste mixer (Inter-Mixer IMX-150, KM Tech, Korea) was used to carefully mix all components. Then, the pre-mixed composite was thoroughly hand-mixed, kneaded, and folded in the mortar until self-standing sheet could be formed. Finally, the sheet was calendared to form the electrode with the uniform thickness of ~150 um. The electrode sheet was punched into 8 mm disks, thereby the average loading of active materials was ~4 mg cm^{-2}. Electrodes with DMPZ were prepared according to previously published paper.[1] To estimate contribution from carbon black, electrodes with carbon black and PTFE beads (weight ratio of 80 : 20 (wt.%)), prepared by the same method as other electrodes.

4.2. Electrolyte preparation method

Total 8 different electrolyte solution systems were prepared using $LiPF_6$ (Sigma Aldrich, USA) or LiTFSI (Sigma Aldrich, China) lithium salts. Carbonate mixture (EC/DMC/EMC, 1:1:1 v/v) or tetraglyme (TEGDME (Sigma Aldrich, USA)) were used as a solvent. For the preparation of EC/DMC/EMC solution, EC/DMC (1:1, v/v) mixture (Soulbrain Co., Ltd., South Korea) and EMC (Tokyo Chemical Industry Co., Ltd., Japan) were mixed in volume ratio of 1 : 1. For each combination of lithium salt and solvent, 1M and 3M solutions were prepared and utilized in cells. Only for the $LiPF_6$ in TEGDME electrolyte system, 2M solution was prepared instead of 3M due to limited solubility of $LiPF_6$ in TEGDME and its very high viscosity. All materials with battery grade quality were purchased and used.

4.3. Electrochemistry

AQ, DMPZ and DMQA electrodes were tested in CR2032 coin-type half-cells, which were assembled with a lithium foil as the counter and reference electrode in argon-filled glovebox (O_2, H_2O < 0.1 ppm). Thickness of lithium foil was 0.2 mm, and its diameter was 16 mm. Since lithium was utilized as a counter electrode, it was used in excess. Celgard PP film was used as a separator. Amount of electrolyte used in the coin cell was 80 uL. AQ electrodes were tested using a commercial carbonate electrolyte, 1M $LiPF_6$ in EC/EMC/DMC (PANAX ETEC, South Korea). DMPZ electrodes were tested with the 3M LiTFSI in TEGDME electrolyte. DMQA electrodes were tested using various types of electrolyte system. All electrochemical tests were performed at 25 ℃. Electrochemical performances of AQ and DMQA as the anode (reduction reaction) were investigated in a voltage range of 1.2 V - 3.3 V. Electrochemical performances of DMPZ and DMQA as the cathode (oxidation reaction) were studied in voltage range of 2.5 V – 4.1 V and 2.7 V – 4.4 V, respectively. Cycling performances were evaluated with galvanostatic (dis)charging cycling tests with potential limits at a current density of 50 mAg^{-1} using the battery testing system (WBSC3000L, WonATech, Korea). In addition, rate capability performance was investigated under various (dis)charging rate from 50 mAg^{-1} to 1200 mAg^{-1}.

The symmetric all-organic full cell was assembled with two DMQA electrodes. The DMQA electrode without any pre-treatment was used as the anode. Whereas, the DMQA cathode was cycled in the voltage range of 2.7 V – 4.4 V for 20 cycles for its activation process and then utilized as the cathode of the full cell. The precycled DMQA cathodes were carefully harvested from the coin-cells after activation process together with the separators and assembled in symmetric cells immediately with the fresh DMQA anode and necessary addition of electrolyte. For the preparation of the all-organic cell, only one kind of the electrolyte (1M $LiPF_6$ in EC/DMC/EMC) was utilized, which allowed reasonable performance of both cathode and anode. The best performance of the all-organic cell was obtained when a weight ratio between anode and cathode was 1.00 : 1.17. The active material loading of the cathode was around 4 mg/cm^2, and that was ~3,42 mg/cm^2 for the anode. The weight difference of the electrodes could be achieved by control of the electrode thickness. Based voltage profiles of the anode and the cathode, the maximum discharge capacity of the anode and charge capacity of the cathode were expected to be ~1.25 and 1.00 units of the capacity per electrode, respectively. Therefore, so-called the N/P ratio was 1.25 and the performance of the all-organic cell became cathode-limited. In addition, excess of the anode is necessary because of the low initial coulombic efficiency and relatively fast capacity decay of the DMQA anode. The all-organic cell was cycled between 0 V and 3.2 V at a current density of 50 mAh g^{-1}.

4.4. Electrochemical test after re-injection of the electrolyte.

The electrochemical cycling of DMQA electrodes for the one cycle and twenty cycles was performed. Cycled cells were carefully disassembled and cell components including the DMQA cathode, the lithium anode, and the separator were harvested. The remaining electrolyte was thoroughly removed with napkins. Harvested cell components were re-assembled into coin cells with the fresh electrolyte using the cycled Li or new Li anode. The re-assembled coin cells were cycled again, using the same condition with the initial condition.

4.5. Dissolution test of DMQA electrode.

As-prepared and cycled DMQA electrodes were immersed in 2 mL of the electrolyte solvent (EC/DMC/EMC, 1:1:1 v/v) for 12 hours. Then, the solutions were photographed as shown in **Figure S22**. For the solubility estimation, weight of the as-prepared DMQA electrode was measured and then it was immersed in 2 mL of electrolyte solvent (EC/DMC/EMC, 1:1:1 v/v) for 12 hours. Drying was firstly performed inside glovebox during 12 hours followed by keeping in glovebox vacuum chamber for another 2.5 hours. Afterwards the dried electrode was utilized to assemble the coin cell with a lithium anode and then electrochemically cycled. The loss of the discharge capacity was ~11 % compared with the electrode that did not suffer the dissolution test. The capacity loss was translated to the solubility of DMQA in the electrolyte, ~0.071 g/L.

4.6. Ex-situ characterization of the electrodes

The electrodes were carefully harvested from the coin cells at various states of charge for the ex-situ characterization. The harvested electrodes were thoroughly rinsed with DMC (DAEJUNG CHEMICALS, Korea) in the Ar filled glove box to remove residual of the electrolyte. All electrodes were immediately transferred in argon sealed bags and analysed for the Fourier transform infrared spectroscopy (FTIR) without any air exposure. The FTIR spectra were recorded in the ATR IR (400-4000

cm^{-1}) by a Nicolet IS50 FTIR spectrometer (Thermo Fisher Scientific., USA). X-ray photoelectron spectroscopy (XPS) was investigated using X-ray photoelectron spectrometer (K-aplha+, Thermo Fisher Scientific, USA). The electrodes for XPS measurement were transferred into the chamber using a compatible vacuum transfer holder (Thermo Fisher Scientific). Binding energies were calibrated by the C=C bond energy of the C 1s spectra (284.5 eV). The X-ray diffraction (XRD) patterns were collected using a D8 Advanced (BRUKER AXS, USA) with a scan rate of 1° per min, a range of 5-45°, and a Cu Kα1 radiation wavelength of 1.5406 Å.

4.7. Computational details

We conducted ab initio calculations based on density functional theory (DFT) using Gaussian 16 Rev. C.01.[2] Wave functions were approximated with 6-311++g(d,p) basis set, and the exchange-correlation functional was described by Becke-Lee-Yang-Parr (B3LYP) hybrid functional.[3] The polarizable continuum model (PCM) method with dielectric constant (ε) of 50.0 was implemented to implicitly consider the solvation effect of EC/DMC=1:1 used in this work.[4] The atomic partial charge was calculated via Hirshfeld charge analysis using Gaussian program.[2] Molecular structures were visualized with PyMOL 2,4,1.[5] The molecular orbitals were rendered by Gaussian program.[2]

2. Summary of the key battery performances of organic electrode materials

Table S1. Summary of the battery performances of organic electrode materials

Nomenclature	Discharge voltage [V vs (Li/Li^+)][a]	Discharge capacity [$mAhg^{-1}$]	Capacity retention (100 cycles)		Ref.
			[$mAhg^{-1}$][b]	[%]	
Coronene	4.00	40	36.8	92	[6]
**DMQA (cathode)**	**3.85**	**97**	**104**	**100**	**This work**
2,2′-Bi[5-(1,3-dihthiol-2-ylidene)-1,3,4,6-tetrathiapentanylidene] (TTPY)	3.4	168	138	82	[7]
5,10-Dihydro-5,10-dimethylphenazine (DMPZ)	3.49	220	55	25	**This work**
F_2-TCNQ	3.15	110	-	-	[8]
F-TCNQ	3.1	183	-	-	[8]
2,5-Bis(perfluorohexyl)-3,6-dichloro-1,4-benzoquinone (Rf_6-Cl-BQ)	3.1	177	-	-	[9]
2,5-Bis(1,3-dithiol-2-ylidene)-1,3,4,6-tetrathiapentalene (TTP)	3.1	99.8	-	-	[7]
2,5-Bis(trifluoromethyl)-1,4-benzoquinone (CF_3-BQ)	3	162	-	-	[9]
2,5-Bis(perfluorobutyl)-1,4-benzoquinone (Rf_4-BQ)	3	115	-	-	[9]
7,7,8,8-Tetracyanodimethoquinone (TCNQ)	2.9	260	-	-	[8]
Dimethyl-TCNQ	2.9	250	-	-	[8]
2,2′-Bis-p-benzoquinone (BBQ)	2.8	358	-	-	[10]
O-Positioned $Li_4C_8H_2O_6$ (Li_4-o-DHT)	2.85	105	-	-	[11]
1,10-Phenanthroline-5,6-dione (PhenQ)	2.74	231	-	-	[12]
Pyrido[3,4-g]isoquinoline-5,10-dione (PID)	2.71	190	152	80	[13]
Benzo[1,2-b:4,3-b′]dithiophene-4,5-quinone (BDTQ)	2.68	214	-	-	[12]
2,5-Dimethoxy-1,4-benzoquinone (DMBQ)	2.65	312	-	-	[14]
7-Methyl-8-bromo-10-(1′-d-ribityl) isoalloxazine	2.615	106	-	-	[15]
Phenanthraquinone (PQ)	2.61	243	-	-	[12]
1,2,7-Trihydroanthraquinone (1,2,7-THAQ)	2.6	186	160	86	[16]
Nanosheet $Li_4C_8H_2O_6$	2.6	223			[17]
7,8-Dichloro-10-(1′-d-ribityl) isoalloxazine	2.665	102	-	-	[15]
para-Positioned $Li_4C_8H_2O_6$ (Li_4-p-DHT)	2.55	118	-	-	[11]
1,2,5,8-Tetrahydroanthraquinone (1,2,5,8-THAQ)	2.55	180	163	90.6	[16]
2,3,5,6-Tetraphthalimido-1,4-benzoquinone (TPB)	2.535	222	202	91	[18]
Riboflavin (RF)	2.525	105.89	-	-	[15]
Benzo[1,2-b:4,5-b´]dithiophene-4,8-dione (BDTD)	2.52	200	108	54	[13]
Me_2-BQ	2.5	226	-	-	[19]
3,4,9,10-Perylenetetracarboxylic dianhydride (PDA-4N)	2.5	116.3	82.4	70.9	[20]
Benzofuro[5,6-b]furan-4,8-dione (BFFD)	2.48	220	189.2	86	[13]
1,4,5,8-Naphthalenetetracarboxylic dianhydride (NDA-4N)	2.445	141.8	48.8	34.4	[20]
Anthraquinone-1,5-disulfonic acid sodium (AQDS)	2.41	130	120	92.3	[21]
2,7-Bis(lithiooxycarbonyl) pyrene-4,5,9,10-tetraone (LCPYT)	2.39	217	-	-	[22]

Anthraquinone-1-sulfonic acid sodium (AQS)	2.26	173	-	-	[21]
$^{i}Pr_2$-BQ	2.2	128	-	-	[19]
5,7,12,14-Pentacenetetrone (PT)	2.2	236	183	77.5	[23]
1,5-Dihydroanthraquinone (1,5-DHAQ)	2.15	190	145	76.3	[16]
Anthraquinone (AQ)	2.15	195	55	28	**This work**
2,7-Bis(lithiooxycarbonyl)- 9,10-phenanthrenequinone (LCPQ)	2.11	90	-	-	[22]
$^{t}Bu_2$-BQ	2	165	-	-	[19]
2,6-Bis(lithiooxycarbonyl)-9,10-anthraquinone (LCAQ)	1.79	85	-	-	[22]
**DMQA (anode)**	**1.68**	**175**	**142**	**81**	**This work**
Phenazine (PNZ)	1.6	205	120	58.5	[24]
Nanowire croconic acid disodium salt (CADS)	1.6	177	170	96	[25]
Dilithium 2,6-naphthalene dicarboxylate (Li_2-NDC)	0.88	200	-	-	[26]
Dilithium terephthalate (Li_2TP)	0.8	300	-	-	[27]
Calcium terephthalate (CaTPA)	0.8	200	155	78	[28]
Dilithium 4,4′-biphenyldicarboxylate (Li_2-BPDC)	0.7	221	-	-	[29]

[a] Discharge voltages of the molecules with several distinguished plateaus are represented as an average voltage of the plateaus. [b] “-“ represents no cycling data reported for 100 cycles or poor stability within a few initial cycles.

3. DFT calculations on inductive effect of DMQA

Note S1.

The Gibbs free energy ($\Delta G°_{rxn}$) is related to the cell voltage ($E°_{cell}$) by Nernst equation, $\Delta G°_{rxn} = -nFE°_{cell}$. The calculated Gibbs free energy obtained from combining the contribution of vibrational nodes evaluated from the frequency analyses with the computed internal energies can be directly converted to the cell voltage as below:

$$\Delta\varepsilon° = \Delta\varepsilon°_{\mathrm{abs}} - \Delta\varepsilon°_{\mathrm{Li,abs}} = -\frac{\Delta G°}{nF} - \Delta\varepsilon°_{\mathrm{Li,abs}} = -\frac{\sum_{\mathrm{product}}\Delta G° - \sum_{\mathrm{reactant}}\Delta G°}{nF} - \Delta\varepsilon°_{\mathrm{Li,abs}}$$

The absolute voltage of standard H^+/H_2 electrode (SHE) is 4.44 V ($\Delta\varepsilon°_{\mathrm{SHE}}$). Since the standard redox voltage of Li^+/Li versus SHE is –3.05 V, we should subtract and add 1.39 V, respectively, from and to the obtained absolute voltage for n-type reactions and p-type reactions (denoted by $-\Delta\varepsilon°_{\mathrm{Li,abs}}$). As a result, we obtained computed cell voltages of DMQA for the n-type reaction (1.89 V and 1.56 V (vs. Li^+/Li) at the 1st and 2nd reduction, respectively) and the p-type reaction (4.22 V and 5.14 V (vs. Li+/Li) at the 1st and 2nd oxidation, respectively). The deviation between the voltages of DMQA for the 1st oxidation (4.22 V (calculated) vs. 4.03 V (experimental)) is attributed to the sophisticated resonances near amine groups and messy complexes consisting of a few of anions and cations according to the literature.[30]

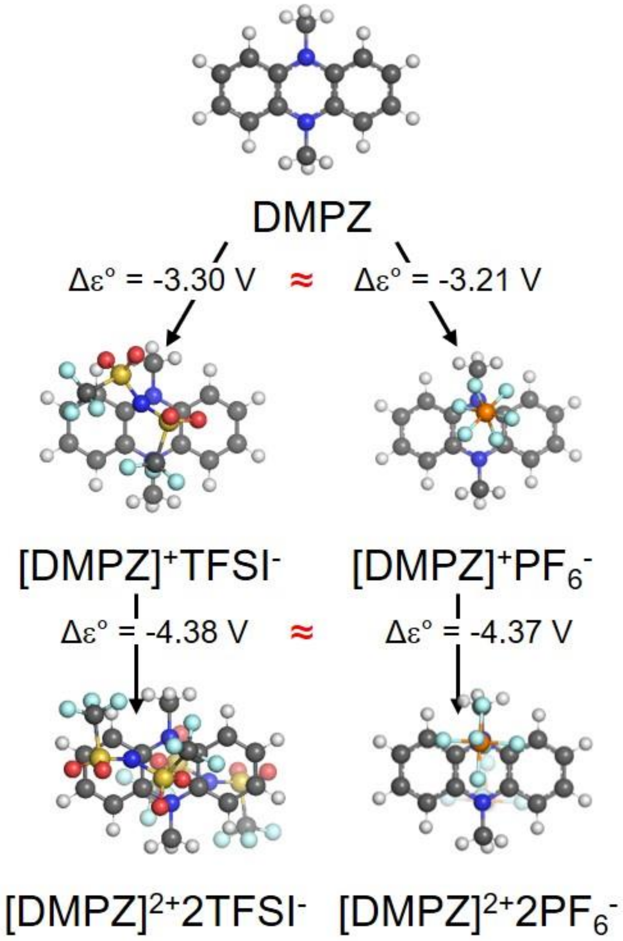

Figure S1. Anion-associated oxidation of DMPZ with $TFSI^-$ and PF_6^- and their corresponding computed cell voltages. The differences in computed cell voltages between $[DMPZ]^+TFSI^-$ and $[DMPZ]^+PF_6^-$ and those between $[DMPZ]^{2+}2TFSI^-$ and $[DMPZ]^{2+}2PF_6^-$ are 0.09 V and 0.01 V, respectively, which are negligible compared with those between $[DMQA]^+$ PF_6^- and $[DMPZ]^{2+}2PF_6^-$ (0.92 V).

Note S2.

To validate the self-reinforced inductive effect of DMQA, we calculated cell voltages of DMQA derivatives (5,12-dimethyl-5,12-dihydroquinolino[2,3-b]acridine-1,8-dione, DMQA-V) as the control group, of which p- and n-type motifs are attached to different carbon rings (**Figure S2a**), and compared the computed cell voltages of DMQA-V with those of DMQA. For the reduction reaction, the computed redox voltages of DMQA (1.89 V and 1.56 V at the 1st and 2nd reduction, respectively) were lower than those of DMQA-V (2.62 V and 2.27 V at the 1st and 2nd reduction, respectively) as shown in **Figure S2b**. In contrast, as shown in **Figure S2c**, for the oxidation reaction, computed cell voltages of DMQA (4.22 V and 5.14 V at the 1st and 2nd oxidation, respectively) were higher than those of DMQA-V (3.30 V and 4.38 V at the 1st and 2nd oxidation, respectively). Our cell voltage calculation convinces that strong self-reinforced inductive effect occurs in DMQA.

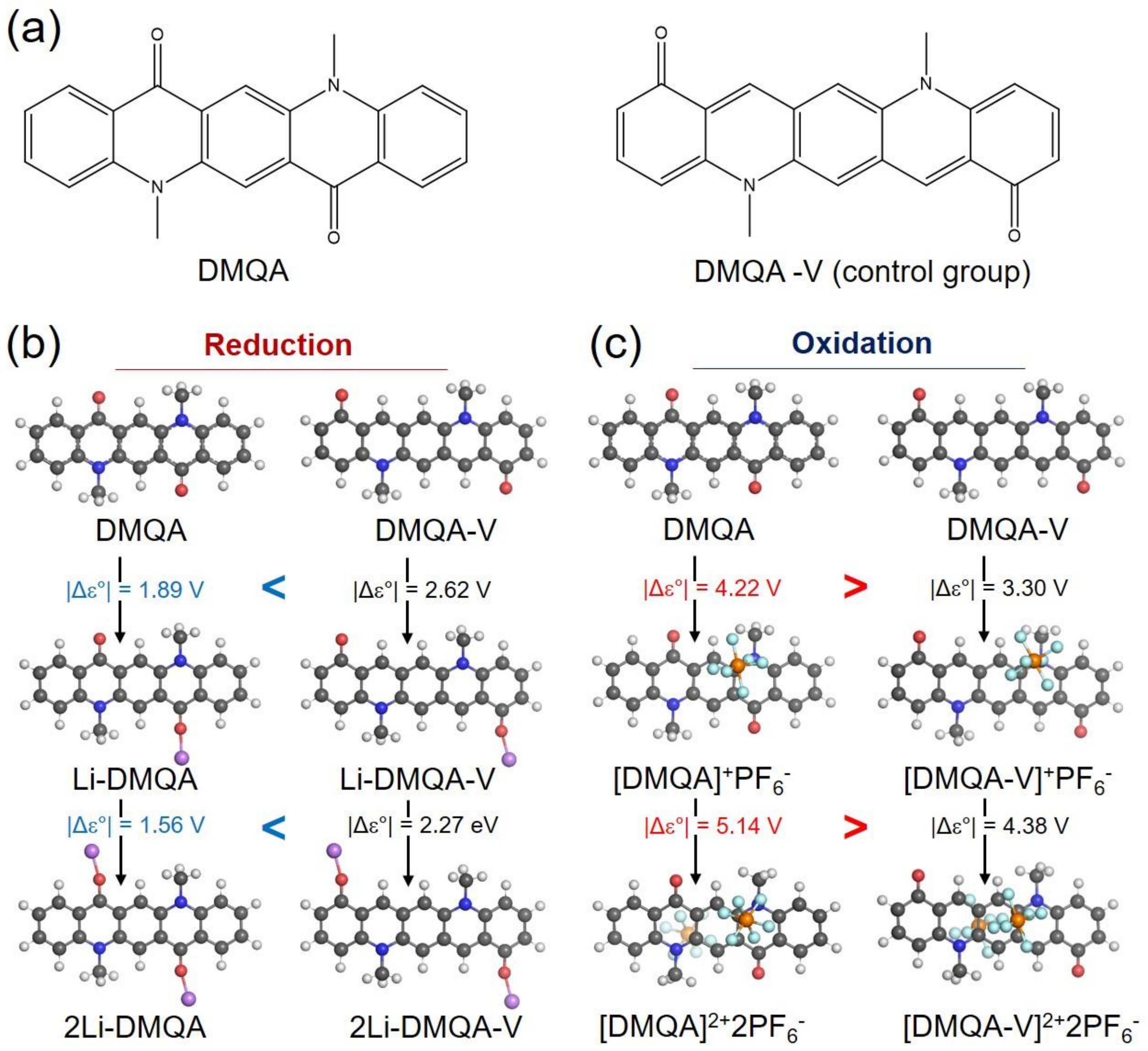

Figure S2. Strong self-reinforced inductive effect of DMQA. (a) Molecular structures of DMQA and DMQA-V. (b) Computed cell voltages (Δε°) of DMQA and DMQA-V during Li^+-coupled reduction. (c) Computed cell voltages (Δε°) of DMQA and DMQA-V during anion-coupled oxidation. Cell voltages (Δε°) were calculated following the method in **Note S1**.

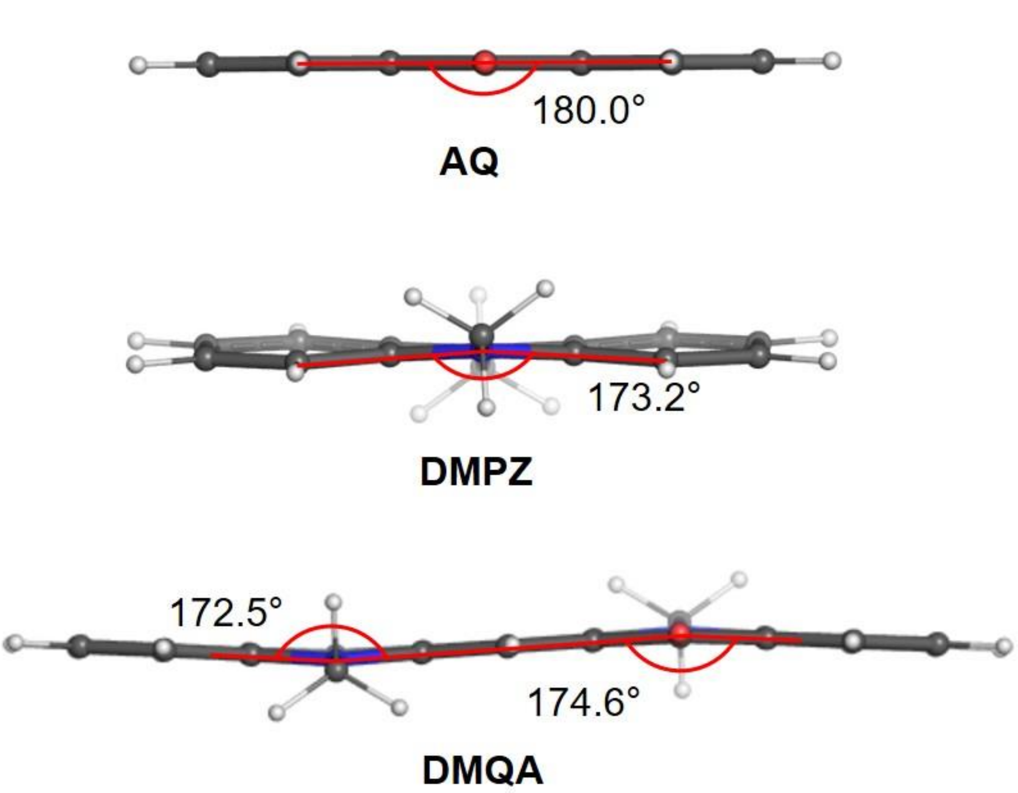

Figure S3. Molecular shapes of AQ, DMPZ and DMQA molecules**.** The linear and bent structures are predicted in the conjugated ring of AQ and DMPZ at the side view, respectively. The conjugated ring of DMQA shows a zig-zag geometry at the side view.

4. Additional tests on electrochemistry of DMQA

Note S3.

Electrochemical performances of the DMQA cathodes were evaluated using various electrolyte systems. The DMQA cathodes using TEGDME based electrolytes showed extremely low coulombic efficiency in the first cycle: ~29% with 3M LiTFSI and < 20% with others. Meanwhile, DMQA with the 1M LiTFSI and 1M $LiPF_6$ in TEGDME electrolytes showed significantly low discharge capacities and inclined voltage curves, which is indicative of capacitive behavior like the carbon additive. After the first cycle, the DMQA cathode showed very limited utilization of DMQA probably due to severe dissolution of oxidized DMQA species into TEGDME. Use of the TEGDME-based electrolyte with high salt concentration also led to fast capacity decay and capacitive response from the DMQA cathode (**Figure S4-S5**). Thus, it seems that all TEGDME based electrolytes are not suitable owing to the significantly deteriorated performance of the DMQA cathode.

Whereas, DMQA cathodes with the carbonate-based electrolyte showed cycling stability (**Figure S6 and Figure S7**). Notable battery performance could be obtained from DMQA using the 3M LiTFSI in EC/DMC/EMC electrolyte (**Figure S6a and b**). However, use of the electrolyte with high salt concentration led to the poor rate performance (see **Figure S8, Figure S9 and Note S4**). The DMQA cathode using the 1M $LiPF_6$ in EC/DMC/EMC electrolyte delivered the best battery performance, including coulombic efficiency, initial discharge capacity, rate capability, and capacity retention. Therefore, 1M $LiPF_6$ in EC/DMC/EMC was selected as the electrolyte for the DMQA cathode.

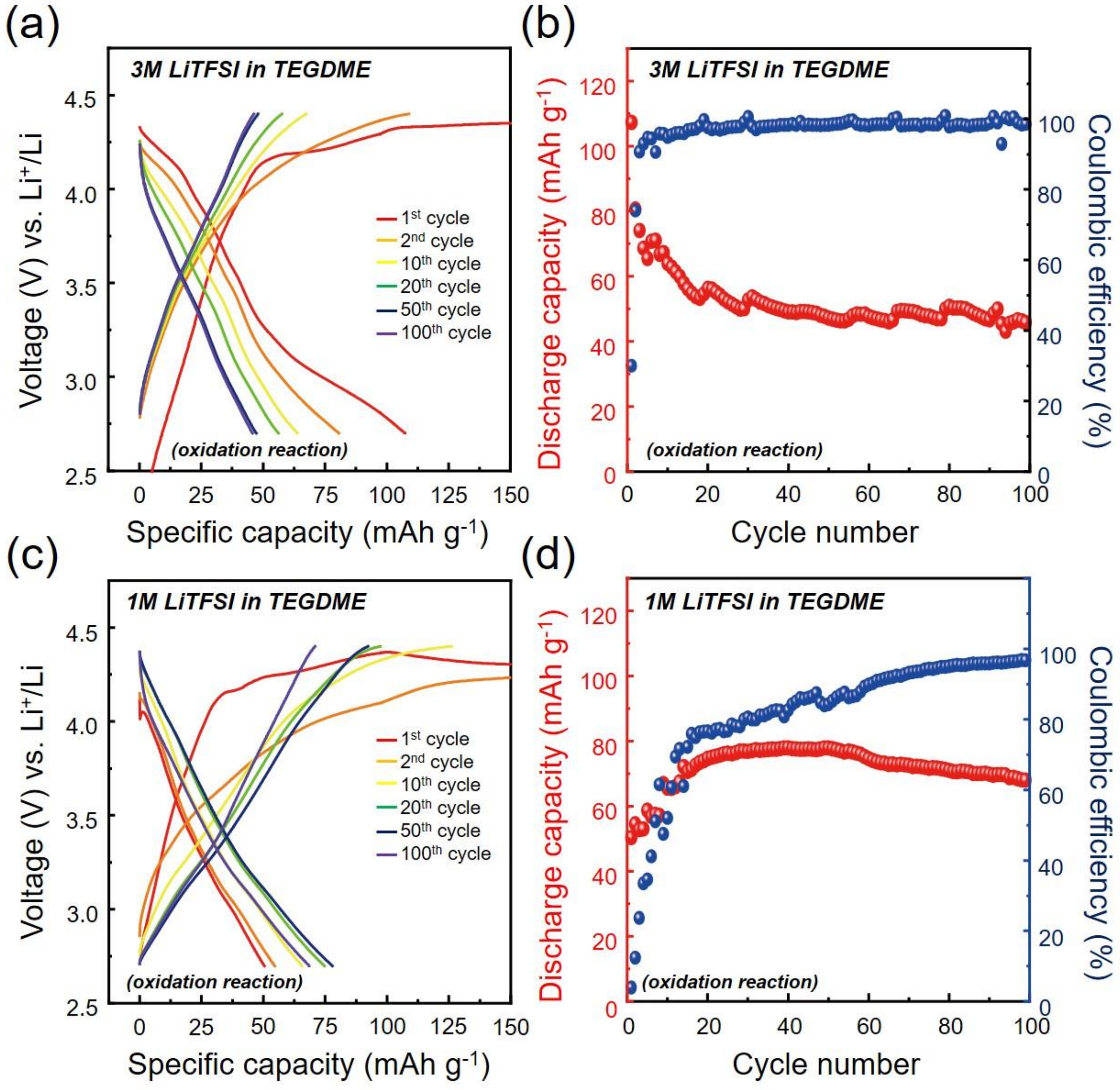

Figure S4. The electrochemical properties of the DMQA cathode were investigated in the voltage range of 2.7 – 4.4 V (vs Li^+/Li) at current density of 50 mA g^{-1} using LiTFSI in TEGDME electrolyte systems. The capacity vs. voltage profile of DMQA cathode using the (a) 3M LiTFSI in TEGDME and (c) 1M LiTFSI in TEGDME electrolyte system. The capacity retention and coulombic efficiency of the DMQA cathode using the (b) 3M LiTFSI in TEGDME and (d) 1M LiTFSI in TEGDME electrolyte system.

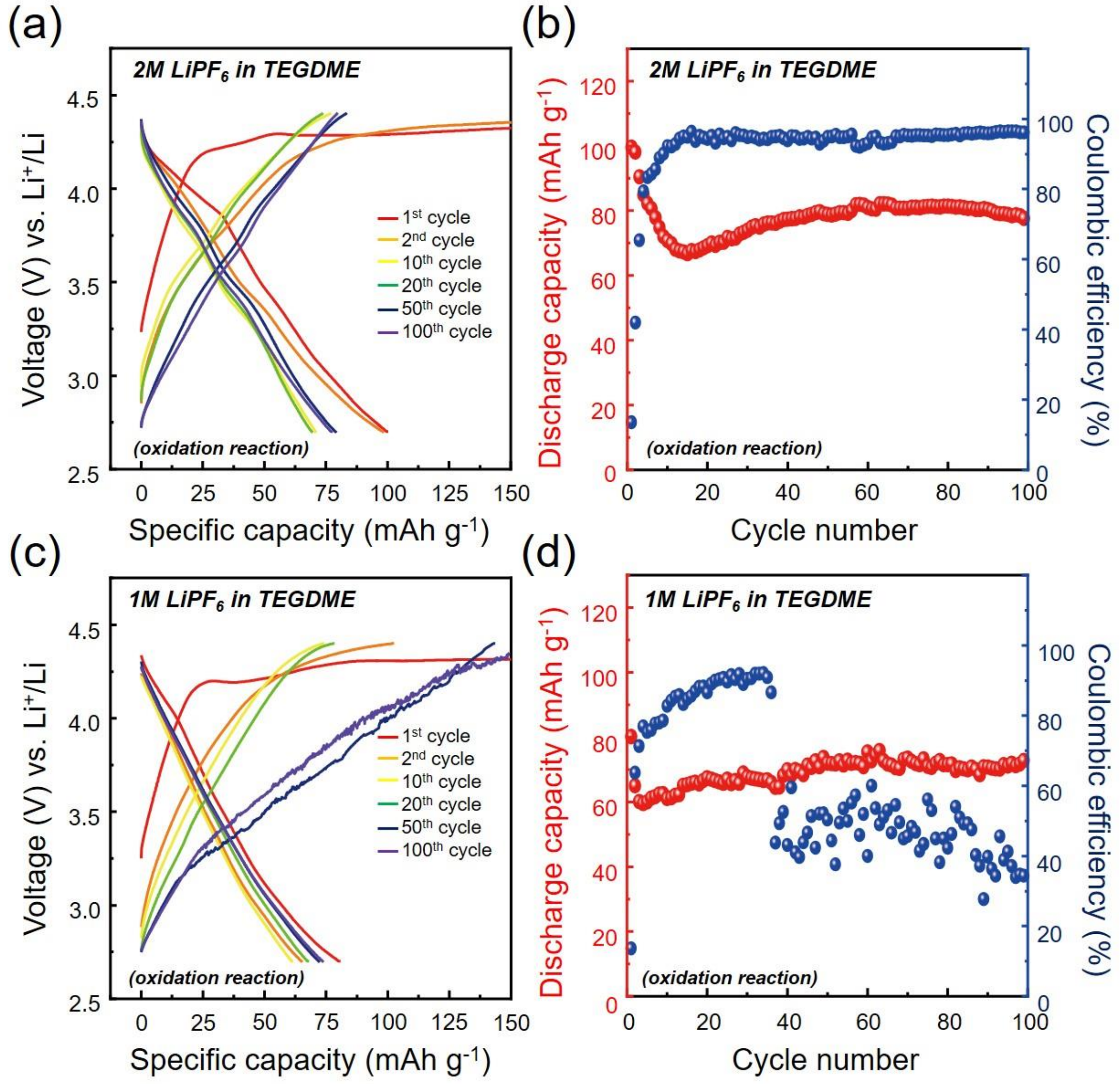

Figure S5. The electrochemical properties of the DMQA cathode were investigated in the voltage range of 2.7 – 4.4 V (vs Li^+/Li) at current density of 50 mA g^{-1} using $LiPF_6$ in TEGDME electrolyte systems. The capacity vs. voltage profile of DMQA cathode using the (a) 2M $LiPF_6$ in TEGDME and (c) 1M $LiPF_6$ in TEGDME electrolyte system. The capacity retention and coulombic efficiency of the DMQA cathode using the (b) 2M $LiPF_6$ in TEGDME and (d) 1M $LiPF_6$ in TEGDME electrolyte system.

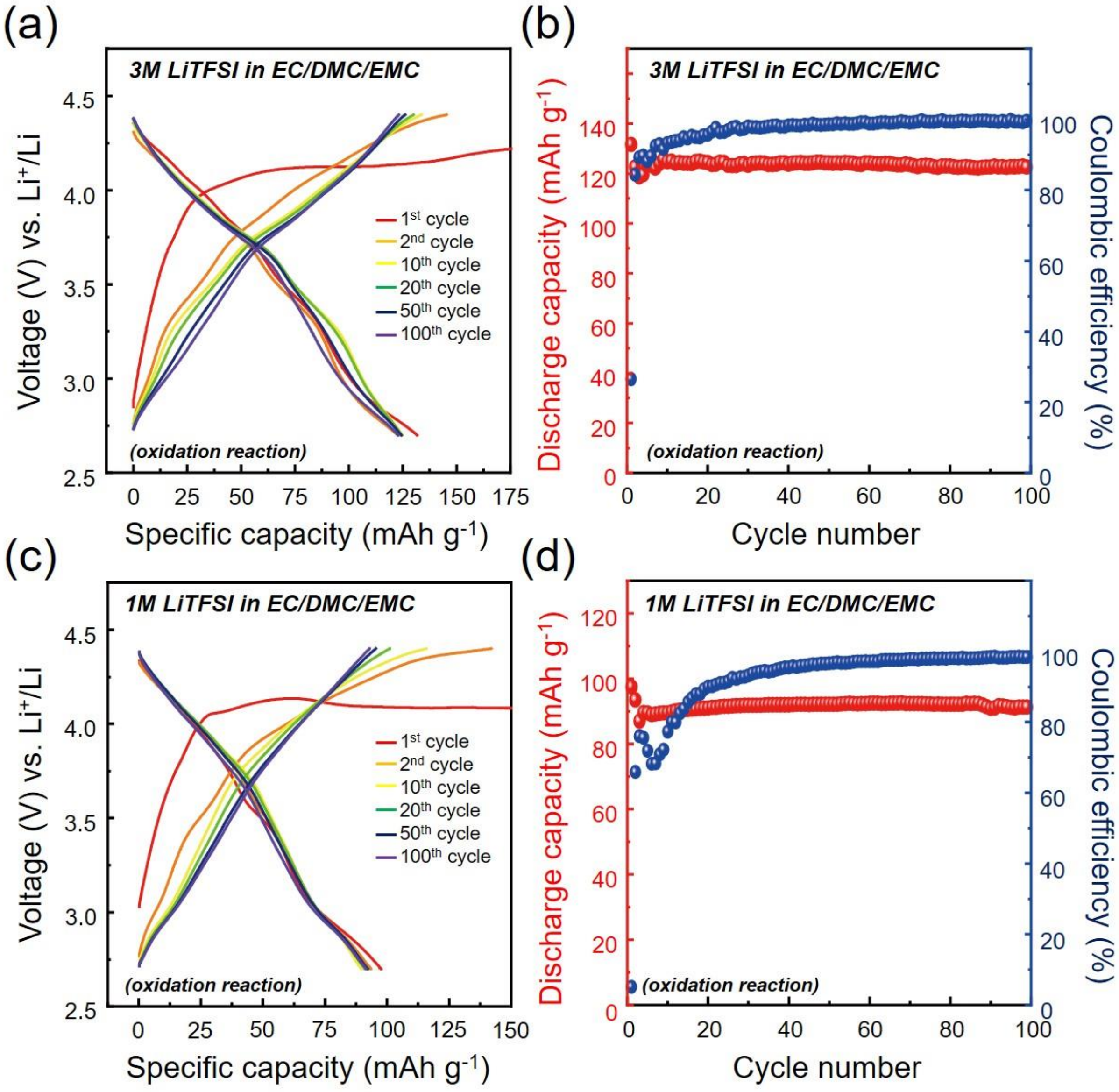

Figure S6. The electrochemical properties of the DMQA cathode were investigated in the voltage range of 2.7 – 4.4 V (vs Li^+/Li) at current density of 50 mA g^{-1} using LiTFSI in the carbonate electrolytes. The capacity vs. voltage profile of DMQA cathode using the (a) 3M LiTFSI in EC/DMC/EMC and (c) 1M LiTFSI in EC/DMC/EMC electrolyte system. The capacity retention and coulombic efficiency of the DMQA cathode using the (b) 3M LiTFSI in EC/DMC/EMC and (d) 1M LiTFSI in EC/DMC/EMC electrolyte system.

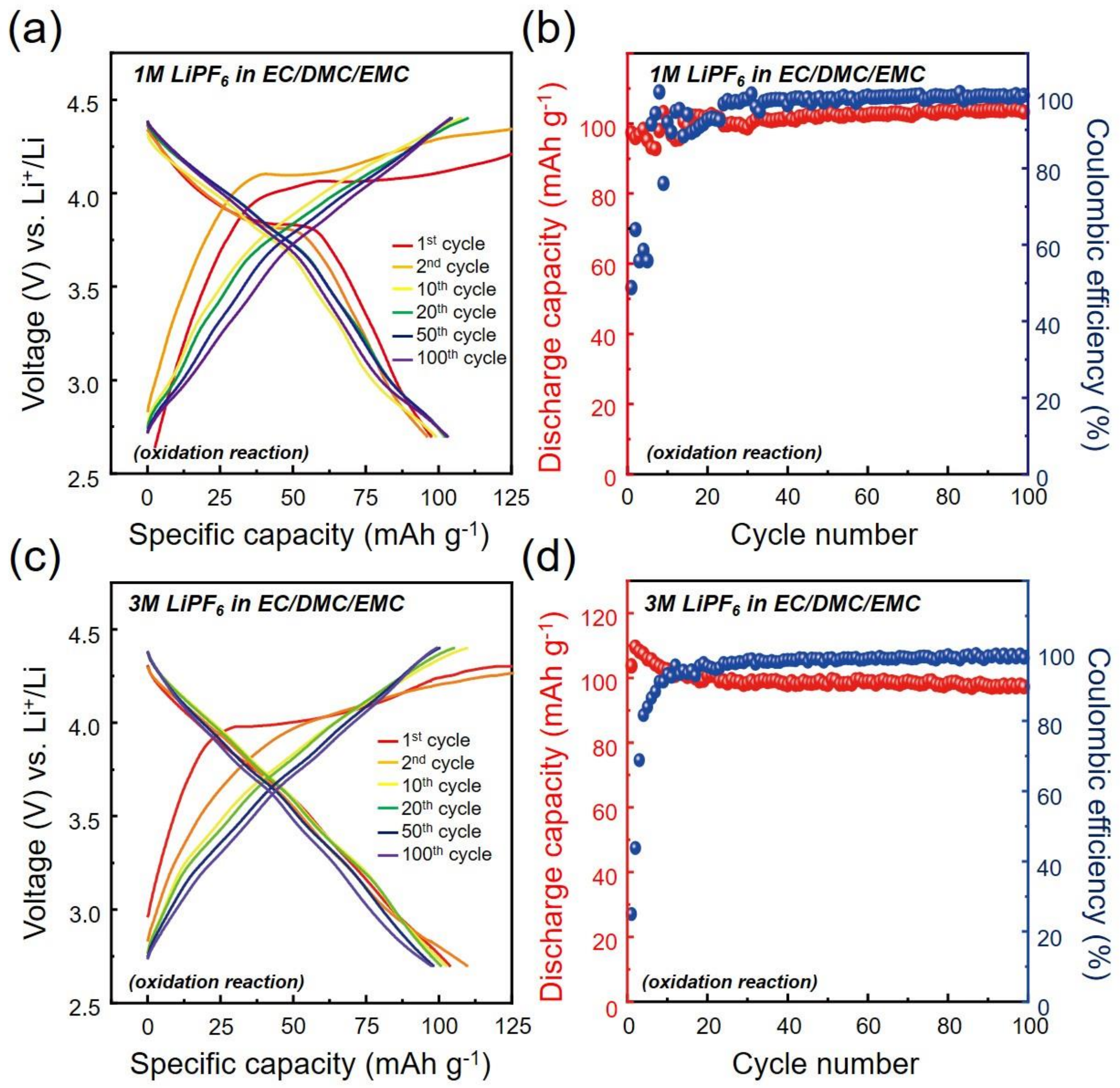

Figure S7. The electrochemical properties of the DMQA cathode were investigated in the voltage range of 2.7 – 4.4 V (vs Li^+/Li) at current density of 50 mA g^{-1} using $LiPF_6$ in the carbonate electrolytes. The capacity vs. voltage profile of DMQA cathode using the (a) 1M $LiPF_6$ in EC/DMC/EMC and (c) 3M $LiPF_6$ in EC/DMC/EMC electrolyte system. The capacity retention and coulombic efficiency of the DMQA cathode using the (b) 1M $LiPF_6$ in EC/DMC/EMC and (d) 3M $LiPF_6$ in EC/DMC/EMC electrolyte system.

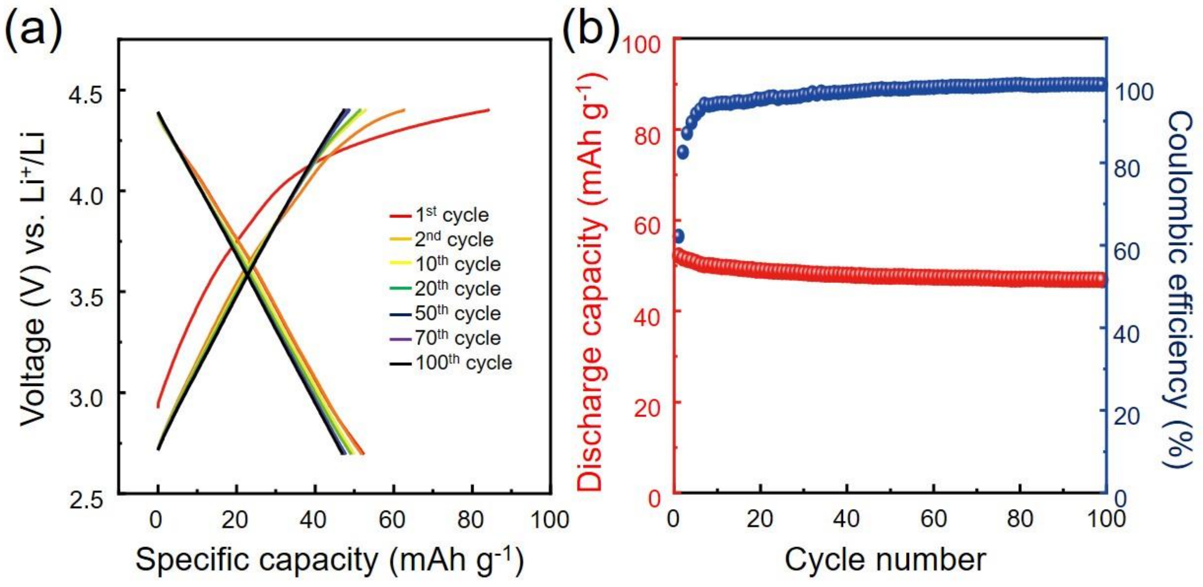

Figure S8. Capacity contribution of Ketjen Black to the capacity of the DMQA cathode in voltage range between 2.7 and 4.4 V (vs Li^+/Li) at current density of 50 mAg^{-1} using the 3M LiTFSI in EC/DMC/EMC electrolyte. (a) Voltage profiles of the Ketjen Black electrode at the representative cycles. (b) Cycle retention of the Ketjen Black electrode.

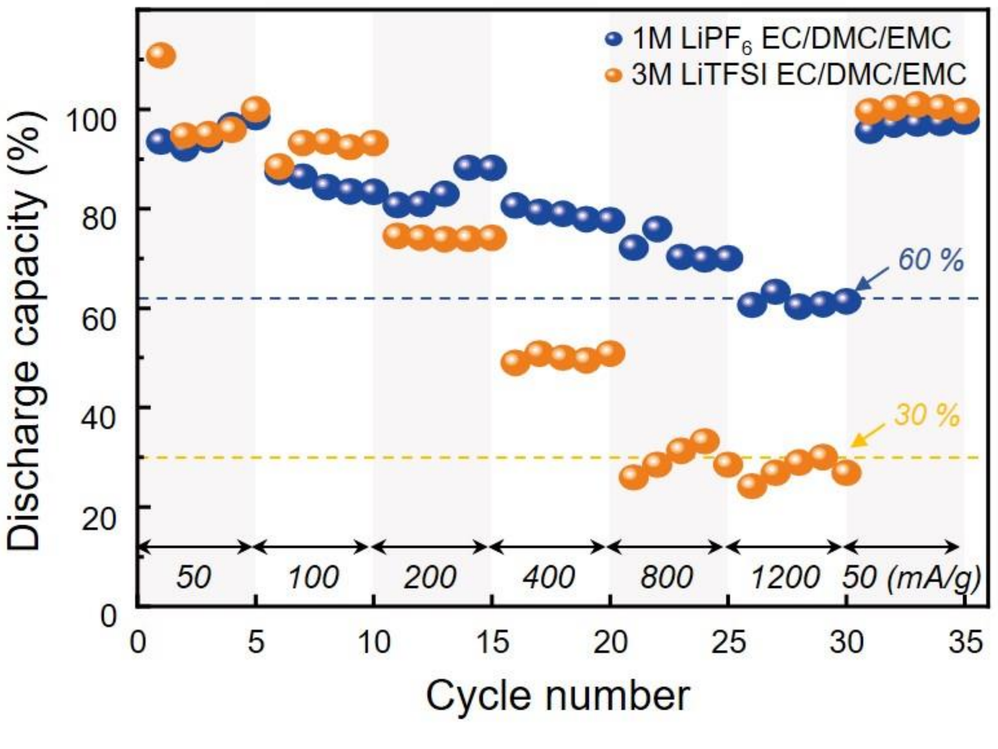

Figure S9. The rate capability of DMQA cathodes between 2.7 and 4.4 V with (orange) 3M LiTFSI in EC/DMC/EMC and (blue) 1M $LiPF_6$ in EC/DMC/EMC electrolytes.

Note S4.
The DMQA cathode with the 3M LiTFSI in EC/DMC/EMC electrolyte delivered discharge capacity of ~132 mAh g^{-1} in the first cycle and ~123 mAh g^{-1} in the following cycles. The contribution of the carbon additive was approximately ~50 mAh g-1 (see **Figure S8**). Hence, the capacity of DMQA could be estimated to be between 65 and 75 mAh g^{-1}, which is slightly less than the theoretical capacity for the one electron oxidation reaction of DMQA. Moreover, the voltage profile curves of DMQA cathodes are very similar when using 3M LiTFSI in EC/DMC/EMC or 1M $LiPF_6$ in EC/DMC/EMC electrolytes, thus the one electron reaction occurs during operation of the DMQA cathode.

Table S2. Summarized key battery parameters of DMQA cathodes under the various electrolyte systems.

Oxidation reaction of DMQA						
Electrolyte composition			Discharge capacity at 1st cycle, $mAhg^{-1}$	Capacity retention at 100th cycle, %	Coulombic efficiency at 1st cycle, %	Average discharge voltage, V vs. Li/Li^{+}
Molarity	Salt	Solvent				
1M	LiTFSI	TEGDME	50	136	4	3.33
3M	LiTFSI	TEGDME	107	43	30	3.38
1M	$LiPF_6$	TEGDME	80	88	14	3.40
2M	$LiPF_6$	TEGDME	100	77	14	3.48
1M	LiTFSI	EC/DMC/EMC	98	94	5	3.49
3M	LiTFSI	EC/DMC/EMC	132	93	27	3.53
**1M**	**$LiPF_6$**	**EC/DMC/EMC**	**97**	**106**	**49**	**3.68**
3M	$LiPF_6$	EC/DMC/EMC	104	94	25	3.50

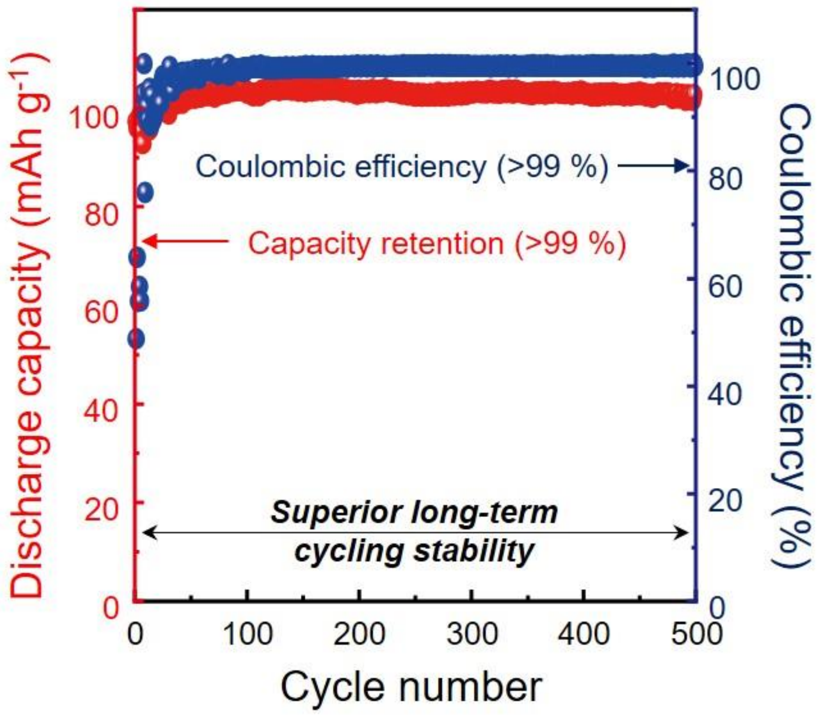

Figure S10. Superior cycling stability of the DMQA cathode can be achieved up to 500 cycles at a current density of 50 mAg^{-1}. (Red) discharge capacity and (blue) coulombic efficiency of the DMQA cathode are presented, which displays notable stability with negligible capacity loss over 500 cycles.

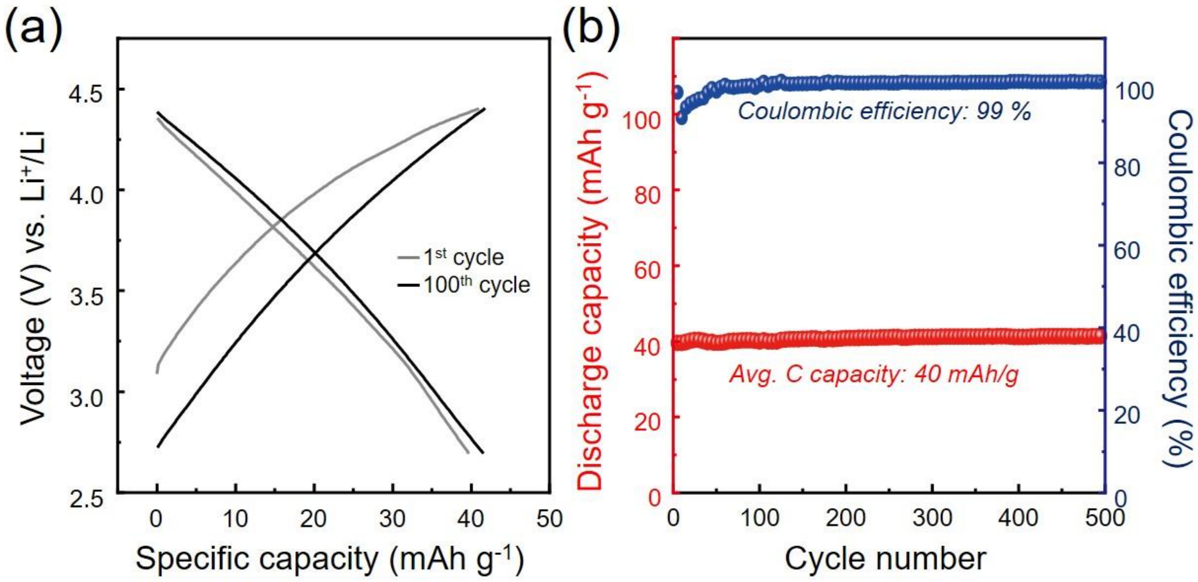

Figure S11. Capacity contribution of Ketjen Black to the DMQA cathode was identified. (a) Capacity vs. voltage profiles of the Ketjen Black electrode (at 50 mAg^{-1} between 2.7 V and 4.4 V, in 1M $LiPF_6$ in EC/DMC/EMC) at the 1st and 100th cycle. (b) Cycling stability of the Ketjen Black electrode.

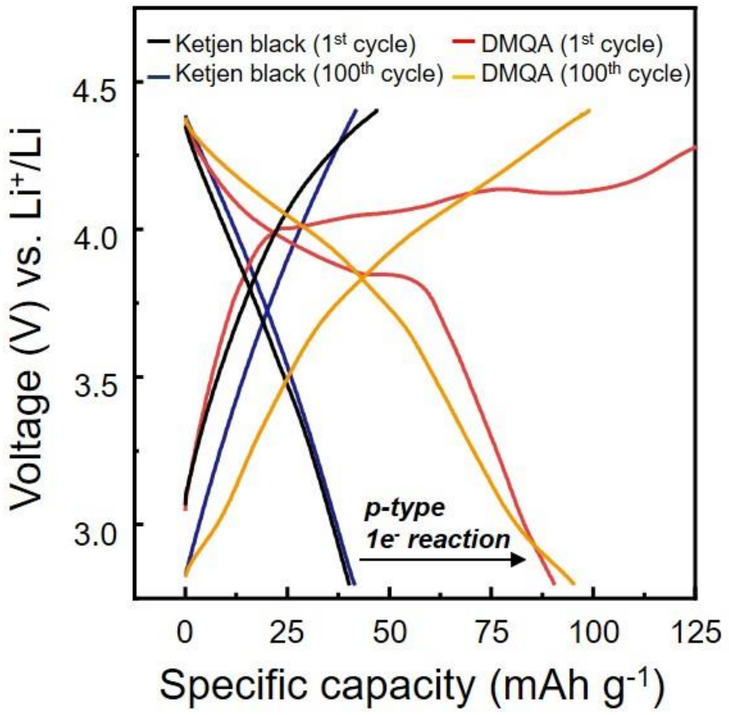

Figure S12. Comparison between capacity vs. voltage profiles of the DMQA cathode and the Ketjen black electrode. Voltage profiles of Ketjen Black and DMQA at the 1st and 100th cycle present the capacity contribution of Ketjen black to the DMQA cathode as cycles are prolonged.

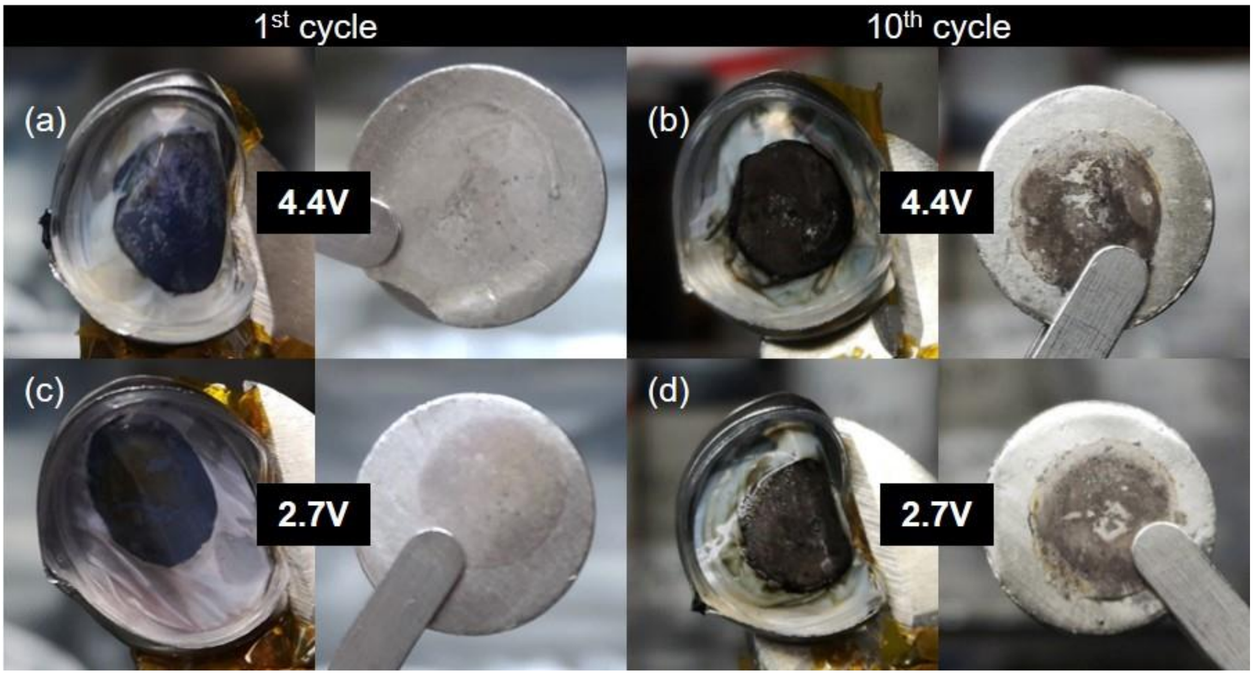

Figure S13. Photographs of the disassembled cells, which represent color of the DMQA cathode side along with that of the separator and electrolyte, and the lithium metal anode. The cells were disassembled at the (a) 4.4 V charged and then (c) 2.7 V discharged state at the first cycle. After 10 cycles, the disassembled cell at (b) 4.4 V charged and then (d) 2.7 V discharged state are also shown.

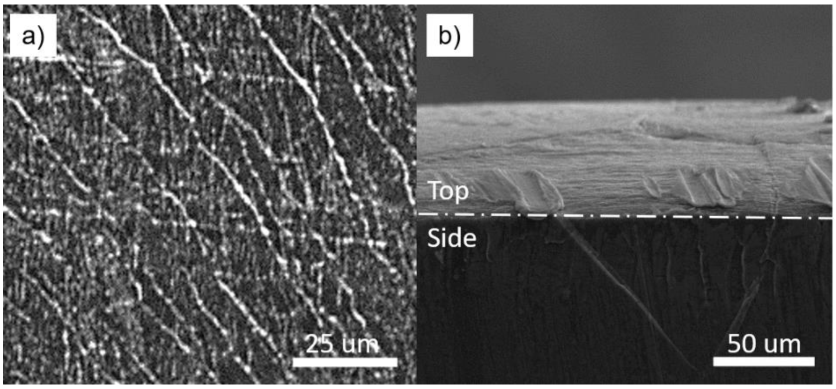

Figure S14. SEM characterization of the lithium anode before cycling. SEM images of top-view (a) and cross-section view (b).

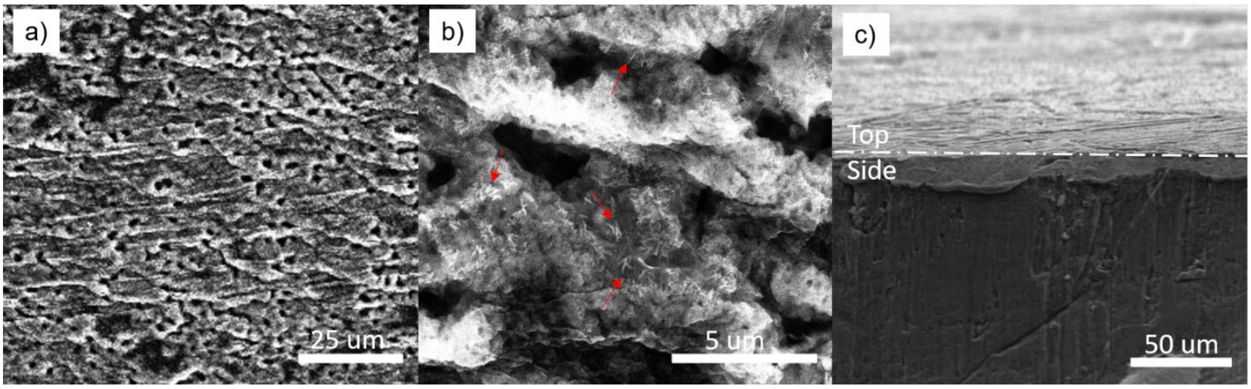

Figure S15. SEM characterization of the lithium anode after the 1 cycle. SEM images of top-view (a and b) and cross-section view (c).

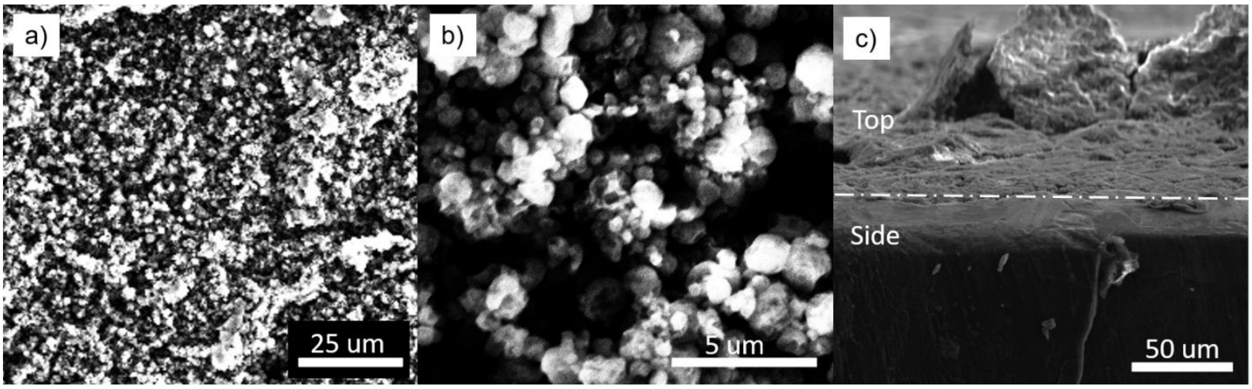

Figure S16. SEM characterization of the lithium anode after 20 cycles. SEM images of top-view (a and b) and cross-section view (c).

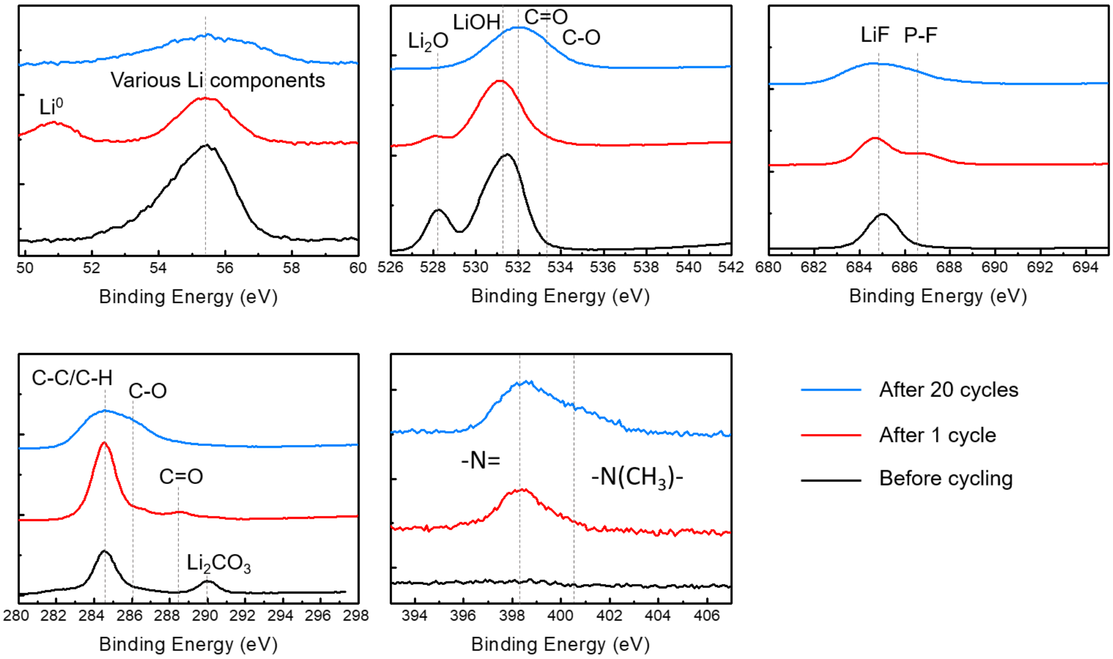

Figure S17. XPS characterization of the lithium anode before cycling, after 1 and 20 cycles.

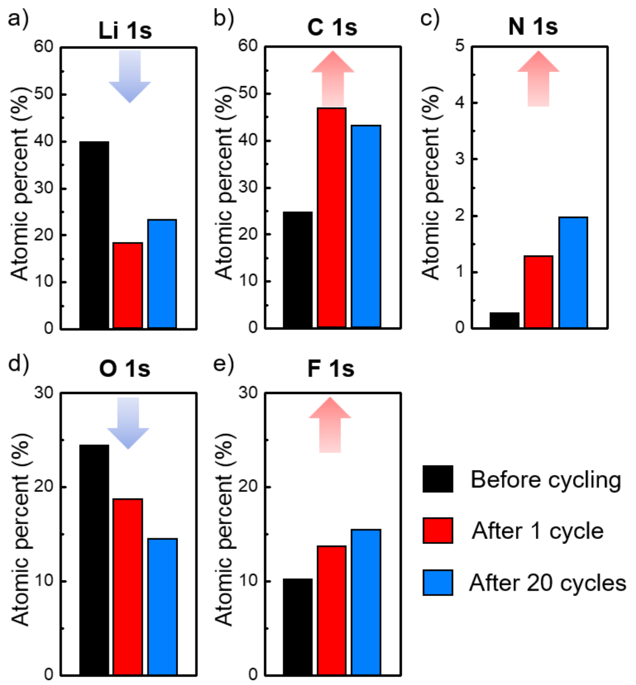

Figure S18. Compositional change of the lithium anode before cycling, after 1 and 20 cycles, which is estimated by the effective XPS spectra area.

Table S3. Binding energies of the compounds that possibly exist in the Li anode.

Component	Binding energy, eV					Based on Ref
	Li1s	C1s	N1s	O1s	F1s	
Li^0	51.1					31
Li_2O	53.5-54			528.3		32
RO-Li	54			531-532		31
LiOH	55			531.4		31
LiF	56				685	31b, 32
Li_2CO_3	56	290		532		31
$LiPF_6$	56.5				686 - 687	31b
C-C / C-H		284.5 - 284.7				32
C-O		285.9		532.5 - 533		32
C=O		288.9		531.5 - 532		32
C-N		285 - 287				33
$-N(CH_3)-$			400.3 - 400.6			This work, 33
-N=			398.5			33

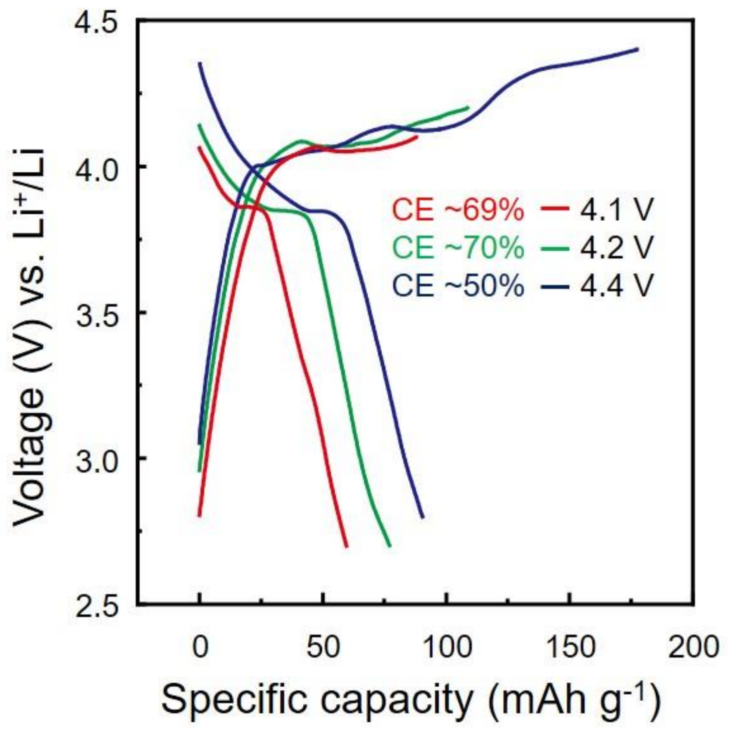

Figure S19. The dependency of the coulombic efficiency (C.E.) and the cut-off voltage condition. Capacity vs. voltage profiles of the DMQA cathode are presented with various charge cut-off voltages (4.1 V (red), 4.2 V (green), and 4.4 V (blue)) at a current density of 50 mAg^{-1}.

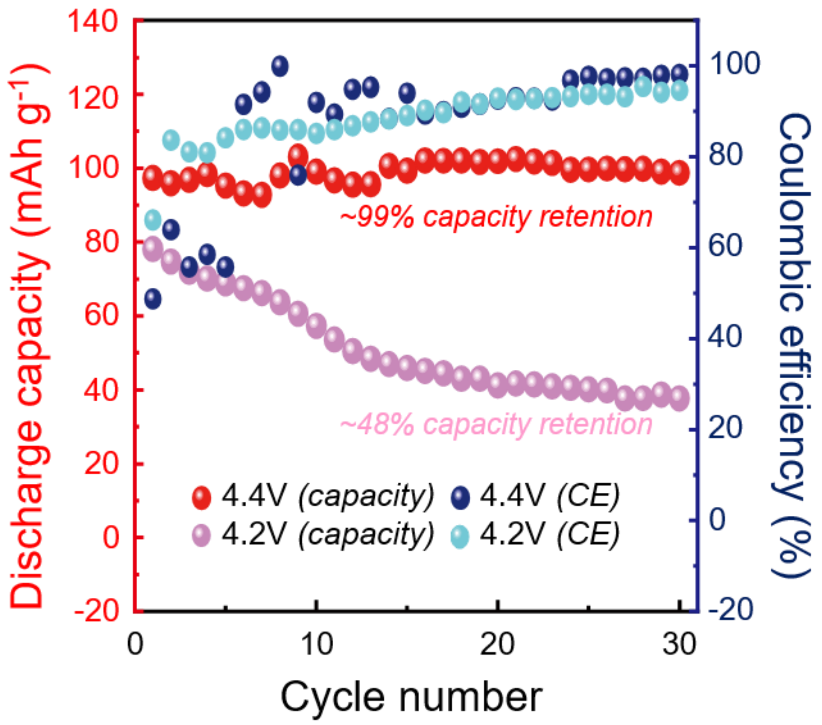

Figure S20. The effect of the charge cut-off voltage on the capacity retention of DMQA cathodes. 4.4 V charge cut-off voltage allows stable cycling performance of the DMQA cathode after activation process at the voltage higher than 4.2 V, highlighting importance of the activation process for the stable cycling performance.

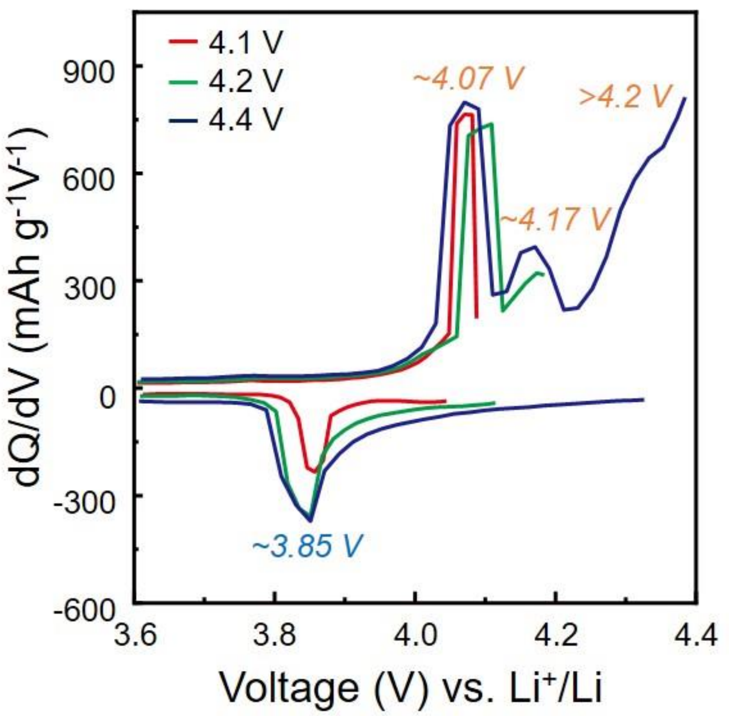

Figure S21. The differential capacity (dQ/dV) plots of the DMQA cathodes were derived from voltage profiles shown in **Figure S20** with various charge cut-off voltages (4.1 V (red), 4.2 V (green) and 4.4 V (blue). Increasing of the charging cut-off voltage to 4.2 V renders appearance of the new anodic peak at ~4.17 V, simultaneously resulting in the increased cathodic peak area at ~3.85 V. Further increase of charging cut-off voltage to 4.4 V led to the irreversible anodic reaction at a voltage higher than 4.2 V.

Note S5.

Supplemental galvanostatic (dis)charging cycling was carried out with the lower cut off condition of 4.1 V and 4.2 V in order to study the origin of low coulombic efficiency at the initial cycle and excellent cyclability at the charge cut-off voltage of 4.4 V. **Figure S19** illustrates that the electrochemical tests with the charge cut-off voltages of 4.1 V and 4.2 V, which shows further increase of the coulombic efficiency from 50% to 70% with the discharge plateau preserved at 3.85 V at the initial cycle. However, the capacity rapidly fades in the following cycles probably due to severe dissolution of DMQA into the electrolyte (**Figure S20**). The dQ/dV curves were also derived from the capacity vs. voltage profiles as depicted in **Figure S21**. The electrochemical cycle with the charge cut-off voltage of 4.1 V reveals one pair of anodic and cathodic peaks corresponding to voltages of 4.07 V and 3.85 V, respectively. Meanwhile, raising charge cut-off voltage up to 4.2 V results in the appearance of the additional anodic peak at 4.17 V and simultaneous increase in the area of the cathodic peak at 3.85 V. It should be noted that the area of anodic peaks is much higher than that of cathodic peaks in both cases with lower charge cut-off voltages of 4.1 V and 4.2 V, which is attributed to the shuttle effect during the charging process.

The electrochemical test with the charge cut-off voltage of 4.4 V reveals emergence of the irreversible anodic peak at the voltage higher than 4.2 V while the other peaks remain unchanged. This irreversible anodic peak can result from the formation of cathode electrolyte interface (CEI), which accounts for the low coulombic efficiency in the initial cycle. The formed CEI could suppress dissolution of DMQA into electrolyte and improve cycling stability. Concurrently, dissolution of DMQA into electrolyte solvent was clearly observed after the 1 cycle, and was not observed after 100 cycles (see **Figure S22**). The sloppy voltage profile, the stable cycling performance, and negligible dissolution after 100 cycles could be attributed to not only formation of the stable SEI and CEI layers but also the electropolymerization of DMQA.

According to the abovementioned discussion, the main origins of low coulombic efficiency at the initial few cycles accompanied by the excellent cycling stability is elucidated by: (1) dissolution of DMQA into the electrolyte followed by the shuttle phenomenon in the initial cycles, (2) gradual formation of the stable SEI and CEI layers, (3) DMQA or $[DMQA]^+$ electropolymerization, which could suppress dissolution of the DMQA during the extended cycles, as evidenced by increase of the coulombic efficiency up to ~100% and negligible capacity decay.

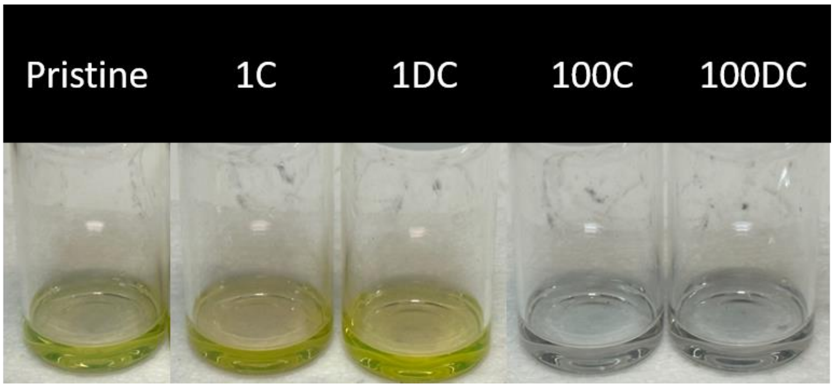

Figure S22. Color of the electrolyte solvent after dissolution test of the as-prepared DMQA electrode, DMQA electrode after 1 cycle, and DMQA electrode after 100 cycles.

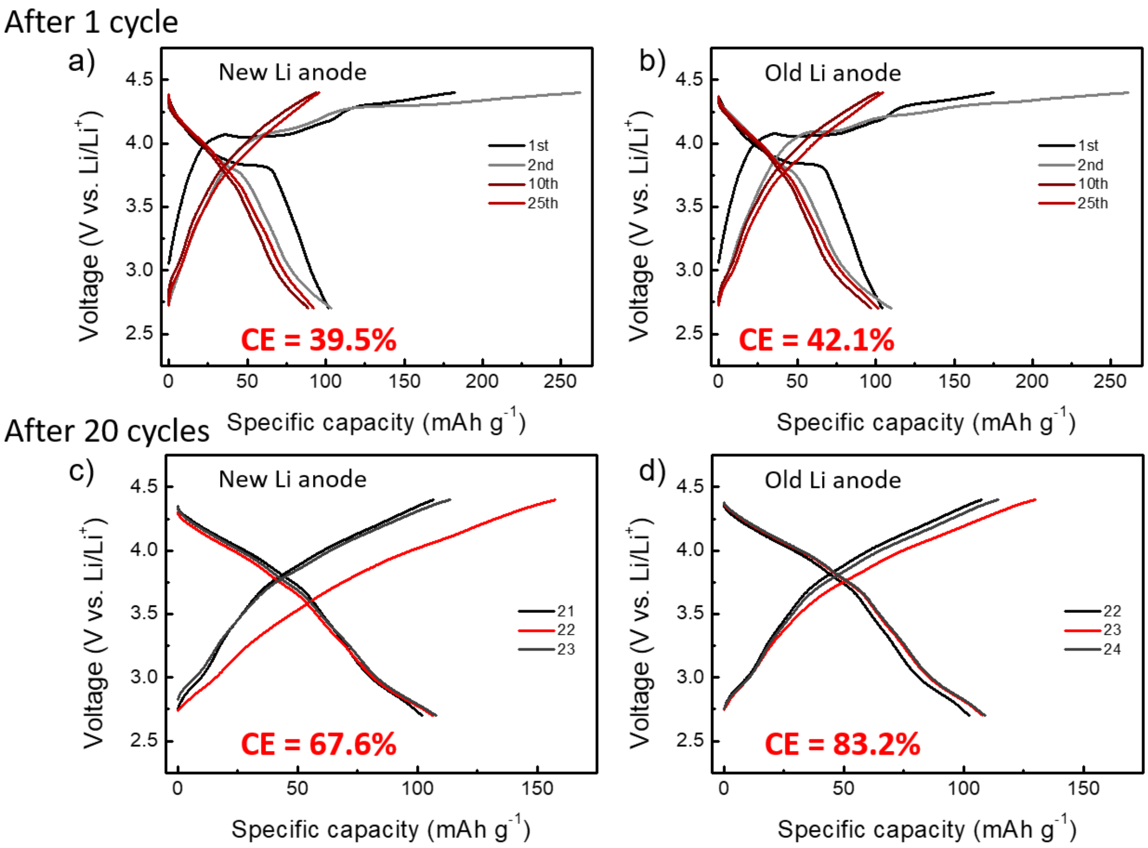

Figure S23. Voltage profiles of the cells after re-injection of the fresh electrolyte. (a and b) after the 1 cycle. (b and c) after 20 cycles.

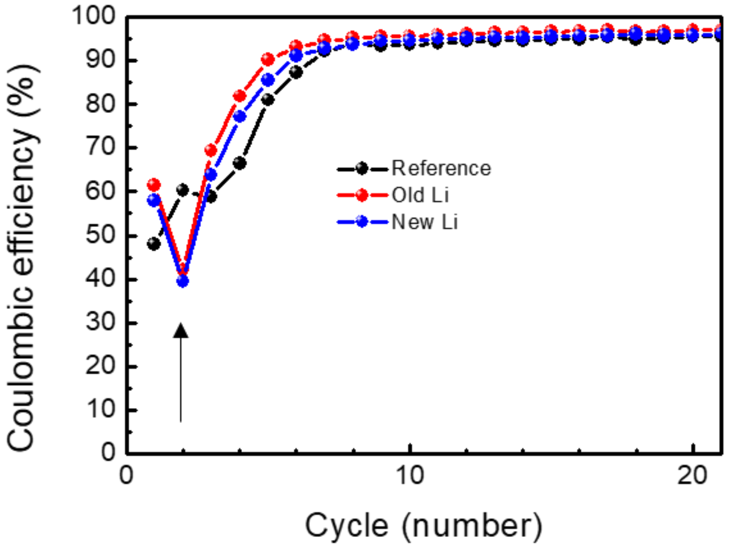

Figure S24. Coulombic efficiency vs. cycle number of the DMQA/Li cells after re-injection of the fresh electrolyte after the 1 cycle.

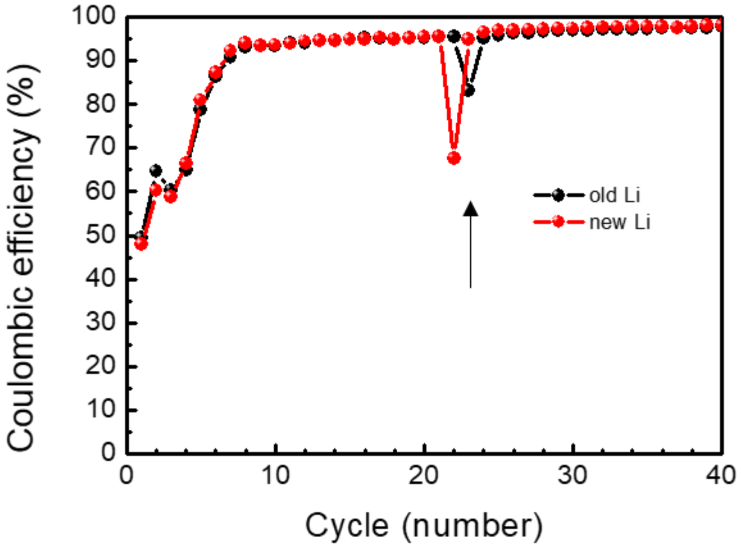

Figure S25. Coulombic efficiency vs. cycle number of the DMQA/Li cells after re-injection of the fresh electrolyte after 20 cycles.

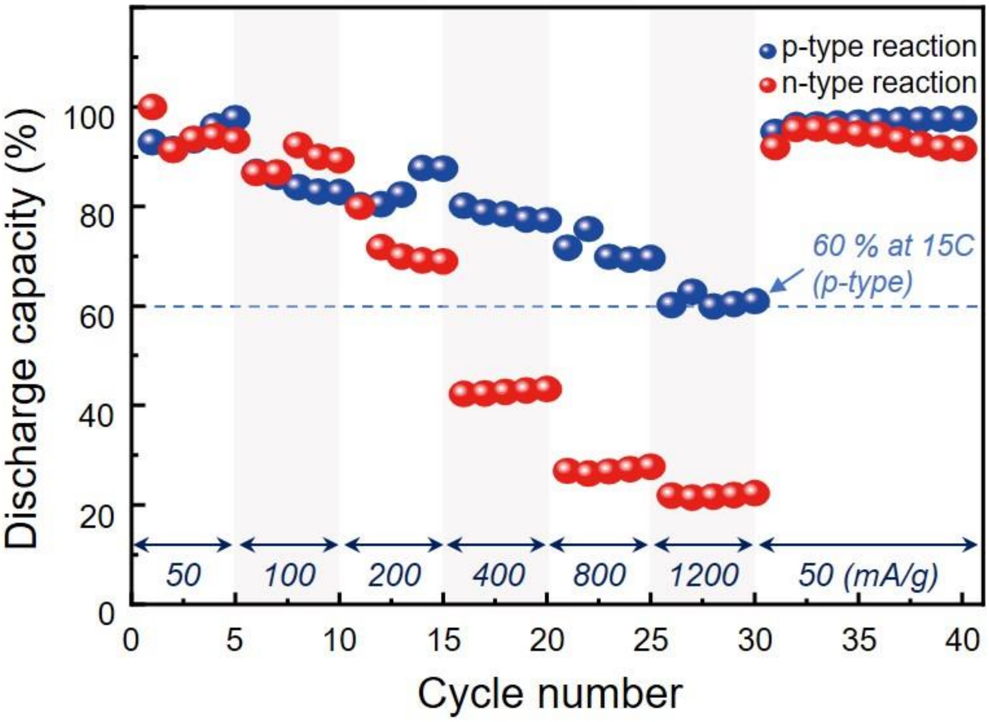

Figure S26. Rate capability of the DMQA (blue) cathode and (red) anode. The DMQA cathode exhibits the fast rate capability of 60% capacity retention at 15 C (1200 mAg^{-1}), indicating its facile electron mobility through conjugated rings of the acene framework. The DMQA anode shows the modest rate capability, 40% capacity retention at a current density of 400 mAg^{-1} (~2.5 C), which is attributed to low ion-conductivity and high viscosity of the electrolyte (3M LiTFSI in TEGDME).

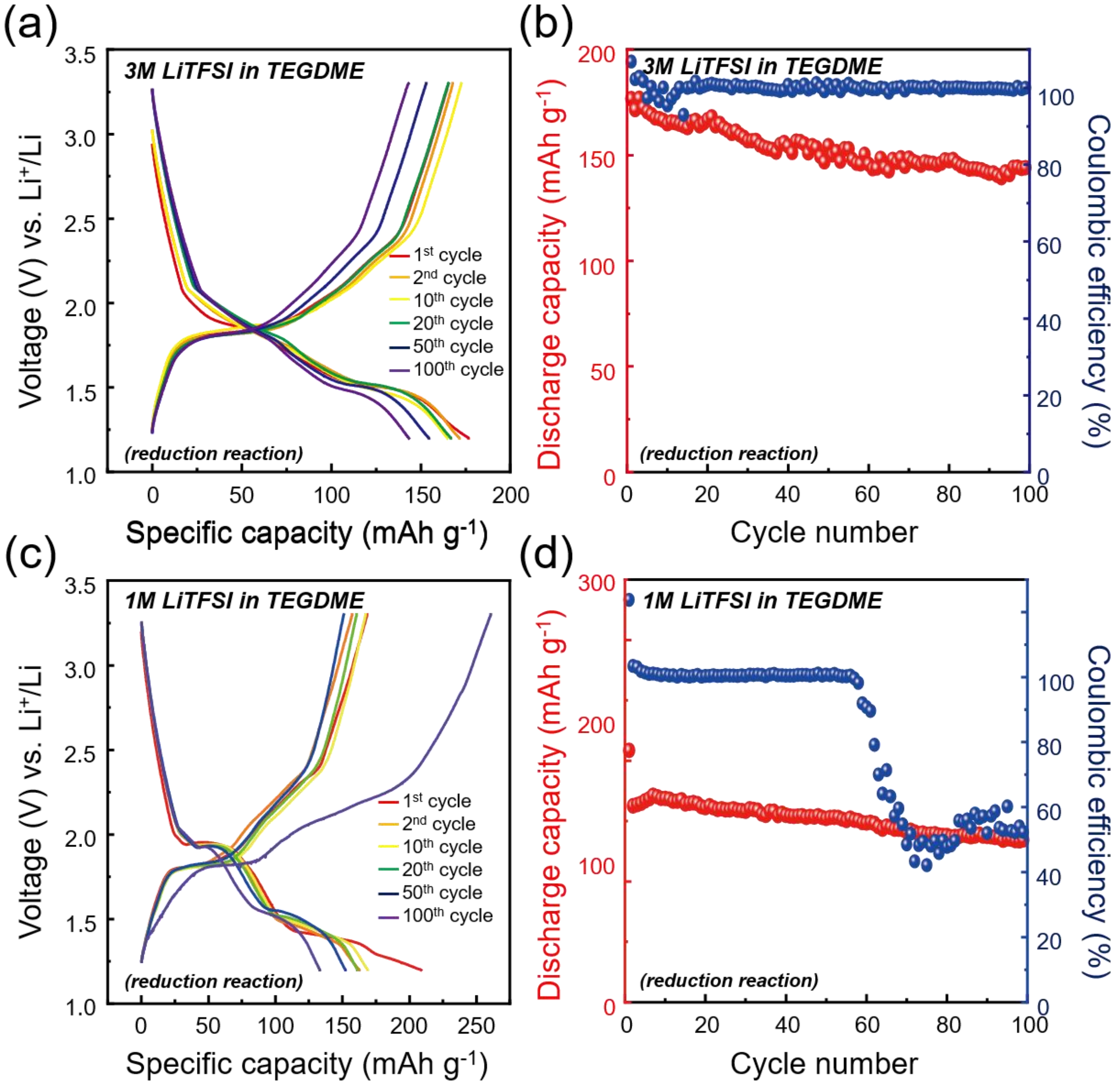

Figure S27. The electrochemical properties of the DMQA anode were investigated in the voltage range of 1.2 – 3.3 V (vs Li^+/Li) at current density of 50 mA g^{-1} using LiTFSI in TEGDME electrolyte systems. The capacity vs. voltage profile of DMQA anode using the (a) 3M LiTFSI in TEGDME and (c) 1M LiTFSI in TEGDME electrolyte system. The capacity retention and coulombic efficiency of the DMQA anode using the (b) 3M LiTFSI in TEGDME and (d) 1M LiTFSI in TEGDME electrolyte system.

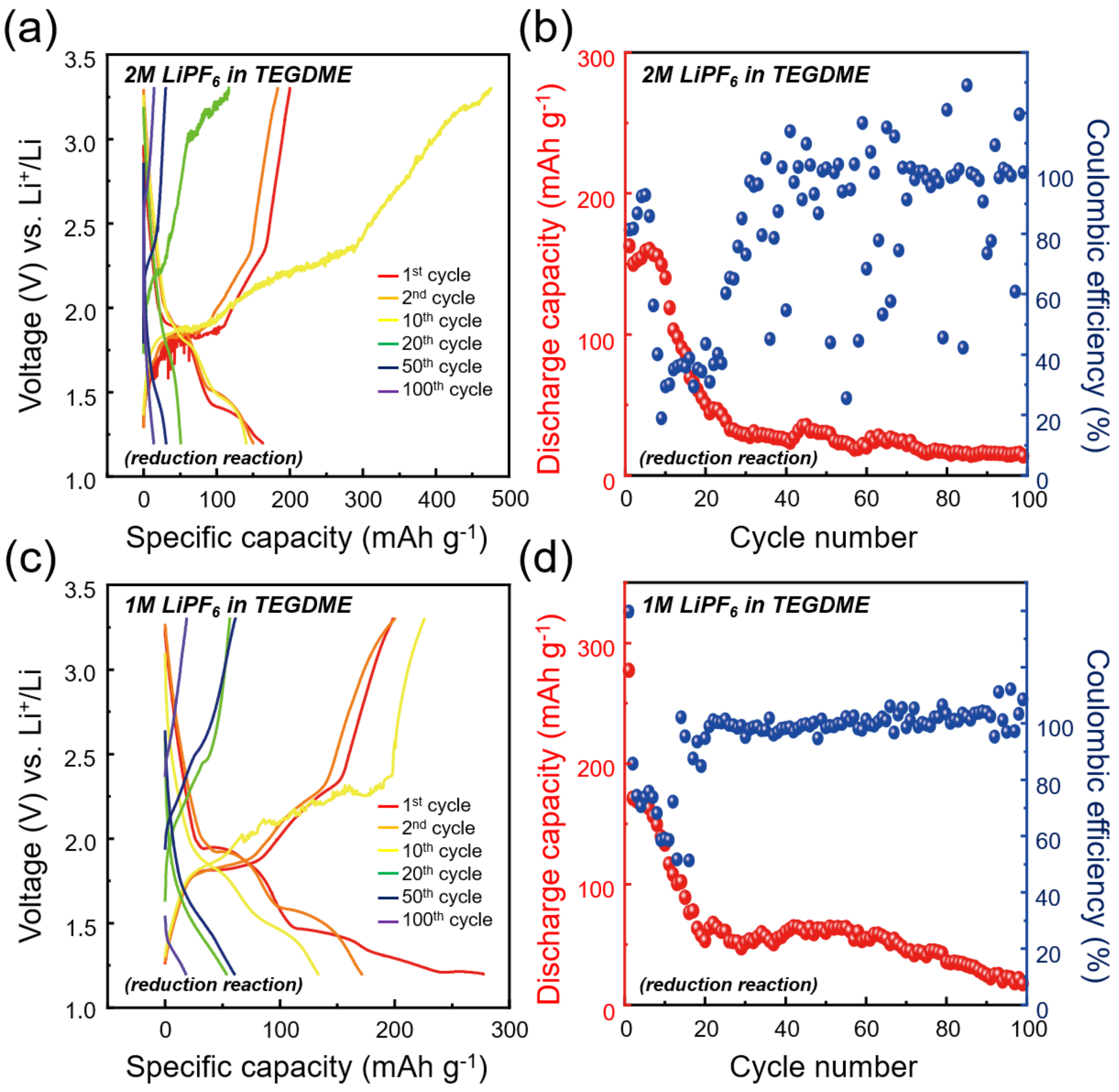

Figure S28. The electrochemical properties of the DMQA anode were investigated in the voltage range of 1.2 – 3.3 V (vs Li^+/Li) at current density of 50 mA g^{-1} using $LiPF_6$ in TEGDME electrolyte systems. The capacity vs. voltage profile of DMQA anode using the (a) 2M $LiPF_6$ in TEGDME and (c) 1M $LiPF_6$ in TEGDME electrolyte system. The capacity retention and coulombic efficiency of the DMQA anode using the (b) 2M $LiPF_6$ in TEGDME and (d) 1M $LiPF_6$ in TEGDME electrolyte system.

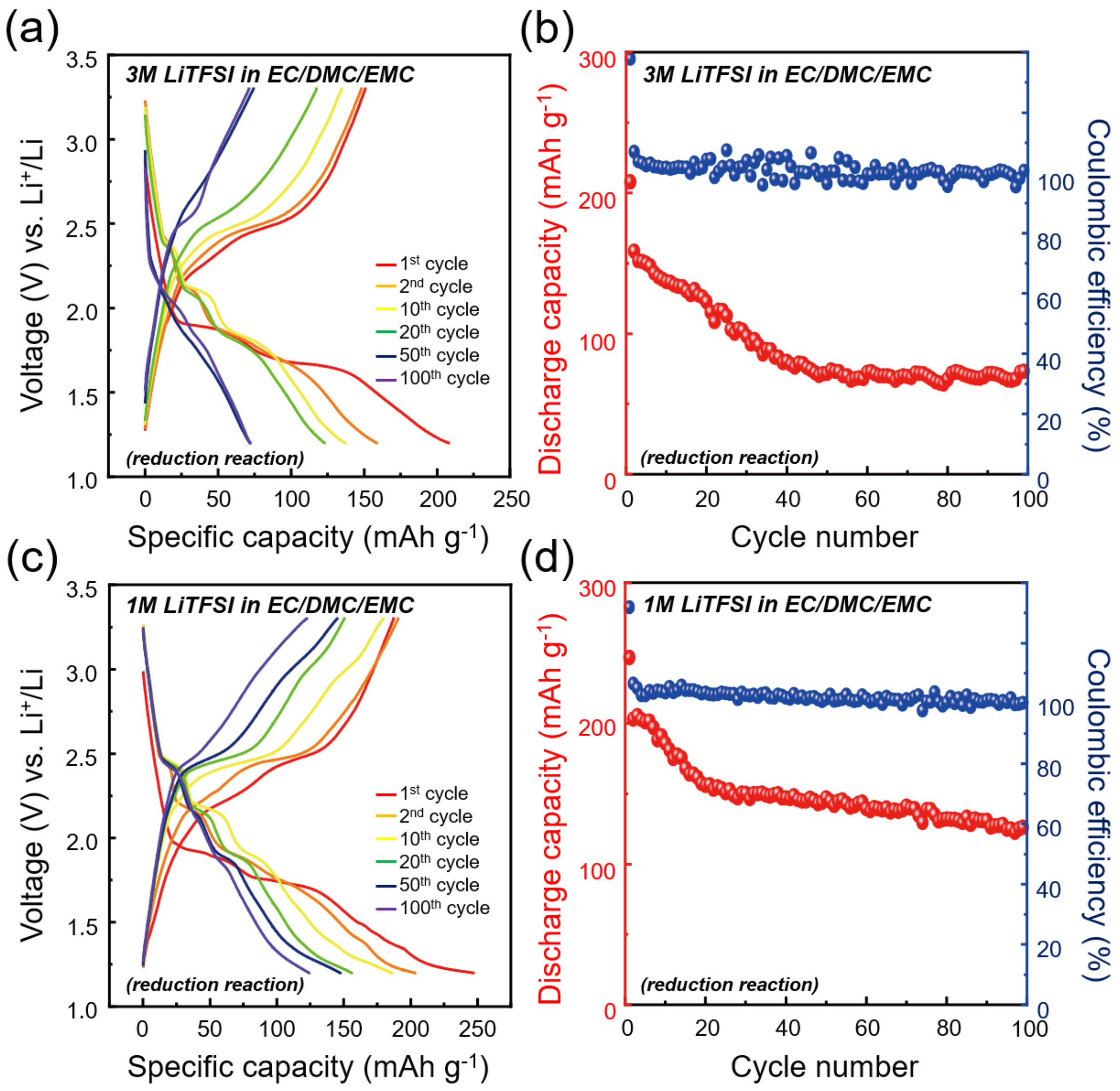

Figure S29. The electrochemical properties of the DMQA anode were investigated in the voltage range of 1.2 – 3.3 V (vs Li^+/Li) at current density of 50 mA g^{-1} using LiTFSI in EC/DMC/EMC electrolyte systems. The capacity vs. voltage profile of DMQA anode using the (a) 3M LiTFSI in EC/DMC/EMC and (c) 1M LiTFSI in EC/DMC/EMC electrolyte system. The capacity retention and coulombic efficiency of the DMQA anode using the (b) 3M LiTFSI in EC/DMC/EMC and (d) 1M LiTFSI in EC/DMC/EMC electrolyte system.

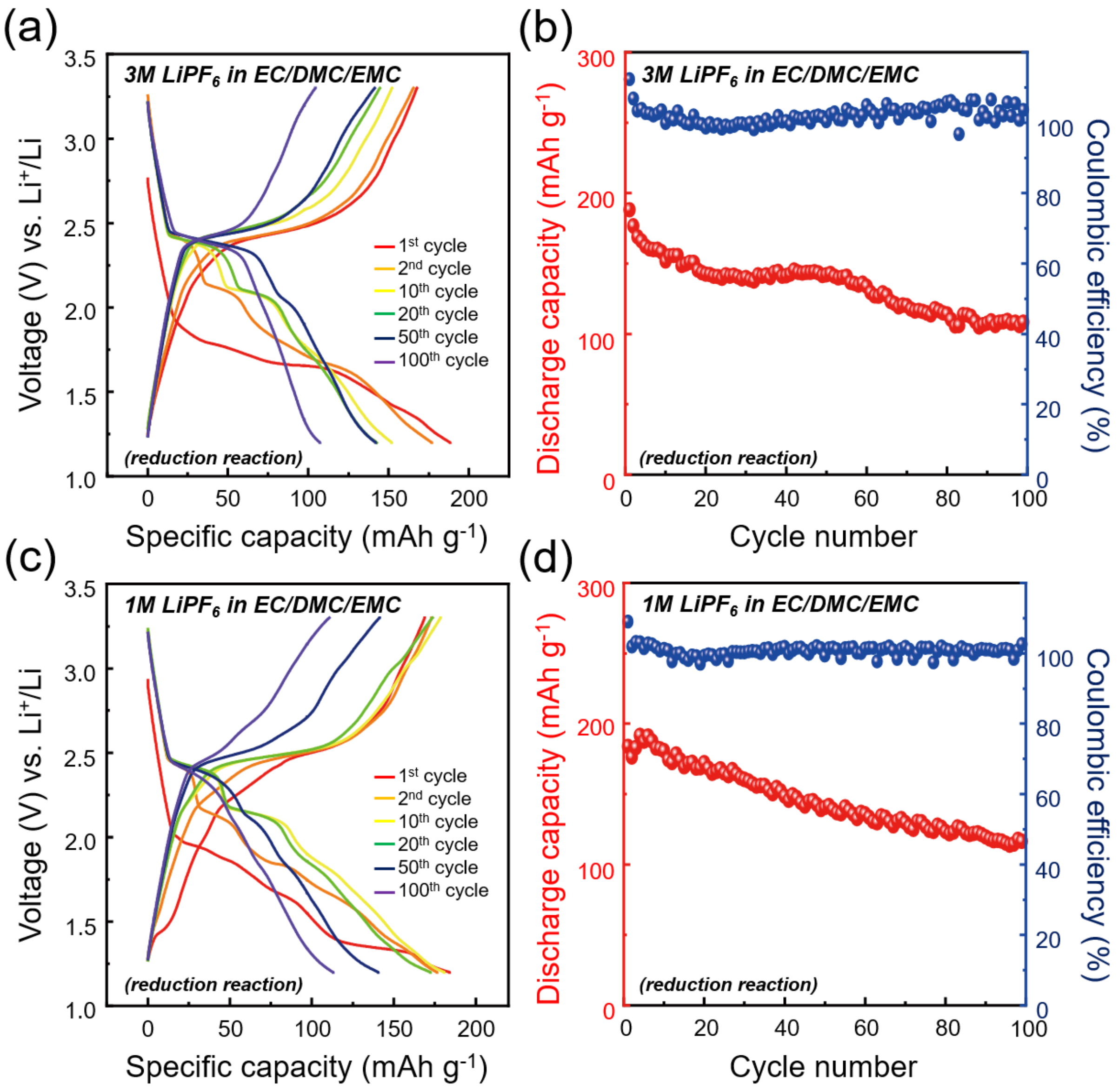

Figure S30. The electrochemical properties of the DMQA anode were investigated in the voltage range of 1.2 – 3.3 V (vs Li^+/Li) at current density of 50 mA g^{-1} using $LiPF_6$ in EC/DMC/EMC electrolyte systems. The capacity vs. voltage profile of DMQA anode using the (a) 3M $LiPF_6$ in EC/DMC/EMC and (c) 1M $LiPF_6$ in EC/DMC/EMC electrolyte system. The capacity retention and coulombic efficiency of the DMQA anode using the (b) 3M $LiPF_6$ in EC/DMC/EMC and (d) 1M $LiPF_6$ in EC/DMC/EMC electrolyte system.

Note S6.

In all carbonate-based electrolytes, the DMQA anode showed significantly deteriorated performance. Cycling stability of DMQA was lower than that in TEGDME-based electrolytes systems followed by severe change of (dis)charging curves upon cycling. The changes in the (dis)charging curve led to higher charging voltage at ~2.5 V, which is undesirable for the anode performance.

The DMQA anodes using the TEGDME-based electrolytes with $LiPF_6$ salt suffered from irreversible reaction during re-oxidation after few initial cycles, which resulted in the decrease in the coulombic efficiency and accessible capacity. The cells assembled with the 1M LiTFSI in TEGDME electrolyte showed significantly lowered cycling performance as coulombic efficiency deteriorated after prolonged cycling (~50 cycles). In addition, with 1M LiTFSI in TEGDME electrolyte DMQA electrode showed overlapping of the second electron reduction with the formation of SEI layer on the surface of conductive additive (additional plateau at below 1.3 V), which complicates utilization of the maximum capacity and lowers charge efficiency in the first cycle.

The best overall performance without critical drawbacks was achieved in the DMQA anode using the 3M LiTFSI in TEGDME electrolyte, which eventually was selected for more detailed evaluation of electrochemical properties of DMQA anode and study about redox -mechanism.

Table S4. Summarized key performance of DMQA anode in various electrolyte solutions

Reduction reaction of DMQA						
Electrolyte composition			Discharge capacity at 1st cycle, $mAhg^{-1}$	Capacity retention at 100th cycle, %	Coulombic efficiency at 1st cycle, %	Average charge voltage, V vs. Li/Li^{+}
Molarity, M	Salt	Solvent				
1M	LiTFSI	TEGDME	171.4	78	124	2.13
**3M**	**LiTFSI**	**TEGDME**	**176.7**	**81**	**107**	**2.09**
1M	$LiPF_6$	TEGDME	171.4	11	140	2.14
2M	$LiPF_6$	TEGDME	162.9	9	81	2.11
1M	LiTFSI	EC/DMC/EMC	203	61	132	2.36
3M	LiTFSI	EC/DMC/EMC	158.7	45	138	2.44
1M	$LiPF_6$	EC/DMC/EMC	184	61	109	2.36
3M	$LiPF_6$	EC/DMC/EMC	188.3	57	112	2.43

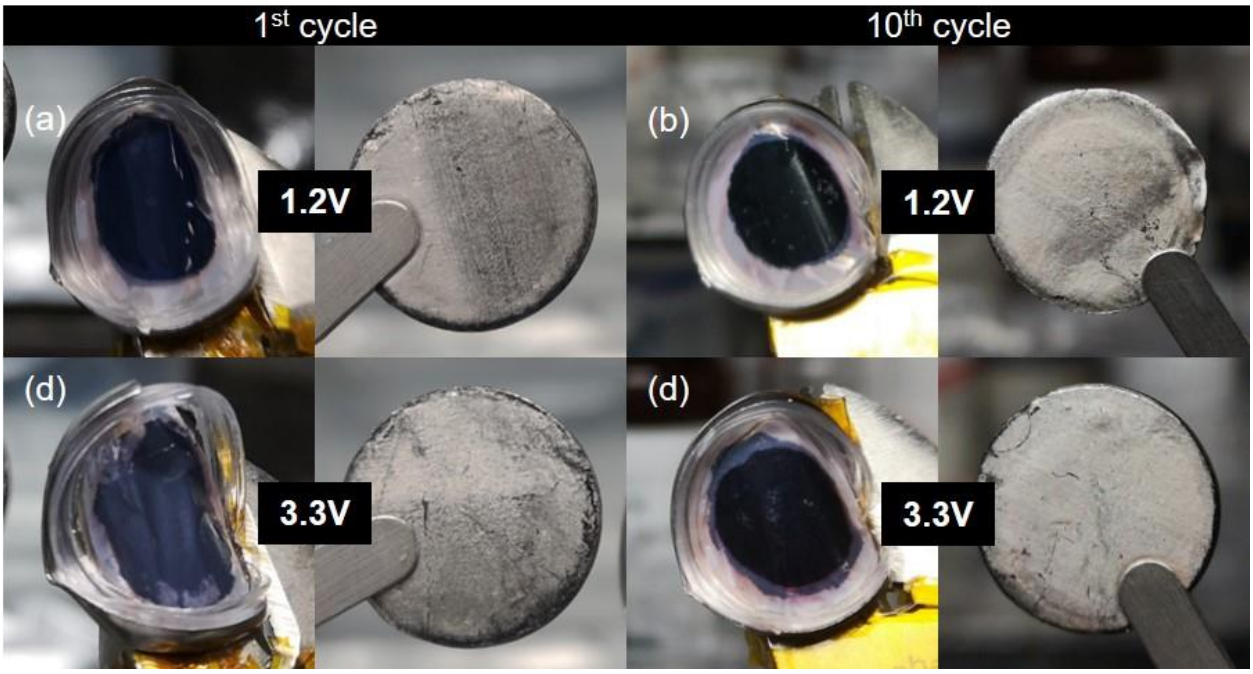

Figure S31. Photographs of the decomposed cells representing colour on the DMQA anode along with those of the separator and electrolyte and lithium metal. The cells were disassembled after (a) discharging to 1.2V and (b) re-charging to 3.3V at the first cycle. Disassembled cells at the (b) 1.2 V discharged and (d) 3.3 V recharged states are also presented. The DMQA anode showed less color change compared with the DMQA cathode.

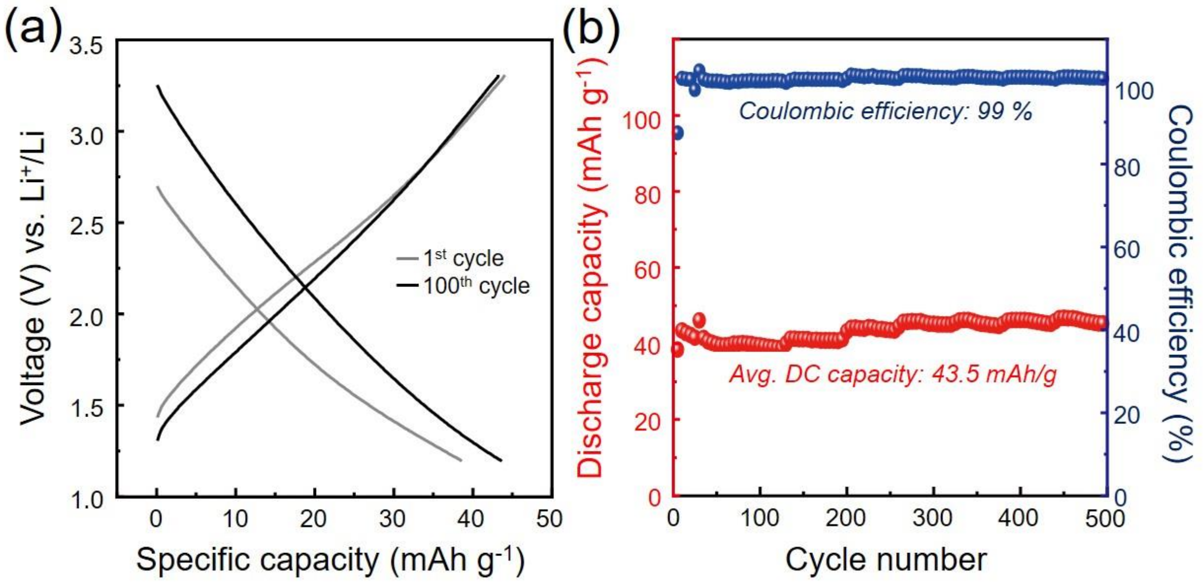

Figure S32. Capacity contribution of Ketjen black to the capacity of the DMQA anode. (a) Voltage profiles of the Ketjen black electrode at the 1st and 100th cycle are displayed. Electrodes were cycled at a current density of 50 mAg^{-1} between 1.2 V and 3.3 V. (b) Cycle retention of the Ketjen black electrode are presented.

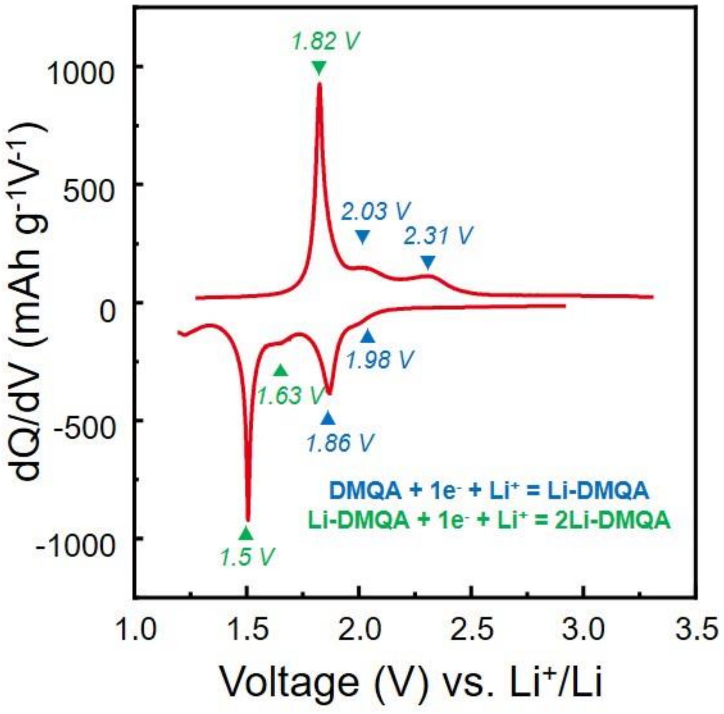

Figure S33. The dQ/dV plots of the DMQA anode during its n-type reaction. The dQ/dV plots were derived from the voltage profile at a current density of 50 mAg^{-1} with a voltage range of 1.2 V – 3.3 V shown in **Figure S27a**.

5. Symmetric cell with DMQA as cathode and anode

Note S7.

We divided the cycle retention graph of all-organic symmetric full cell into 4 regions by the specific possible reasons that are responsible to the capacity decay (**Figure S34a)**. The average voltage and discharge capacity vs. cycle are also shown in **Figure S34b**. We attribute the electrolyte to the main reason for the initial capacity loss of the region I **in Figure S34**). The severe change in the voltage profile of the DMQA anode followed by the capacity decay were observed in the carbonate-based electrolyte (see **Figure S30c,d**), which leads to the dramatic drop of average discharge voltage and the shifts of redox signals of DMQA as shown in the dQ/dV curve (**Figure S34 and S35)**. The capacity retention in the region II (10th-50th cycles) is relatively stable though the average discharge voltage slowly decreases most likely due to the continuous change of the voltage profile change at the anode side (**Figure S35**). Then, the capacity of the full cell is limited by the capacity of the anode after 50 cycles (region III). However, the Faradaic signals from dQ/dV curve still remained in the given voltage range. Finally, after the 65th cycle (region IV), the discharge capacity of the full cell decreased accompanied by the decay of the average discharge voltage. Such loss of the battery performance can result from the severe degradation of the anode and increasing internal resistance (**Figure S30**).

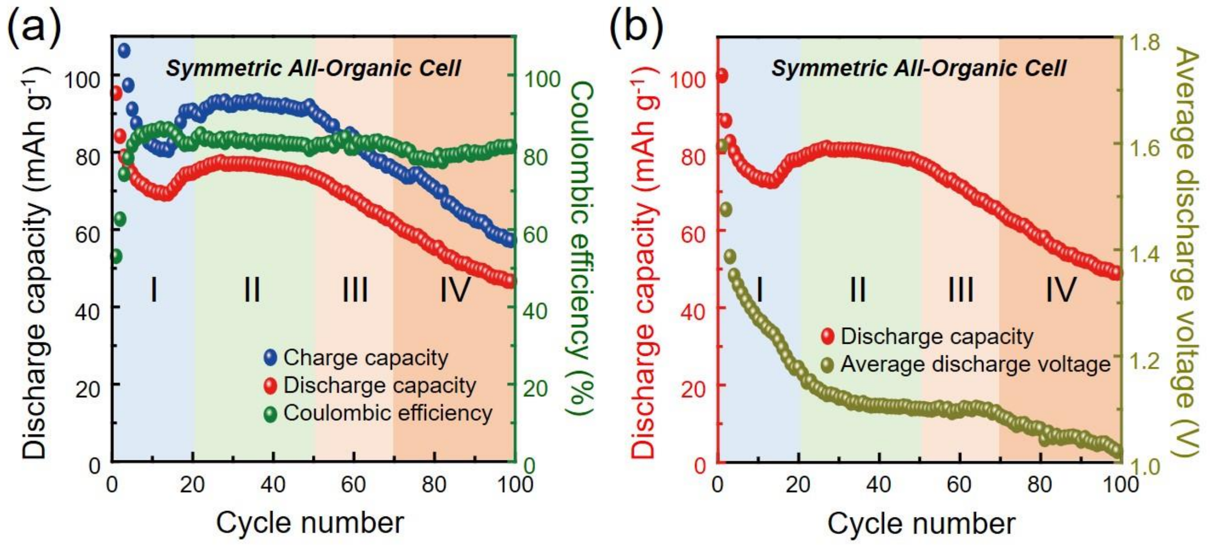

Figure S34. Division of the performance decay of the full organic symmetric cell by 4 regions. (a) cycle retention and (b) capacity and average voltage decay as prolonged cycles.

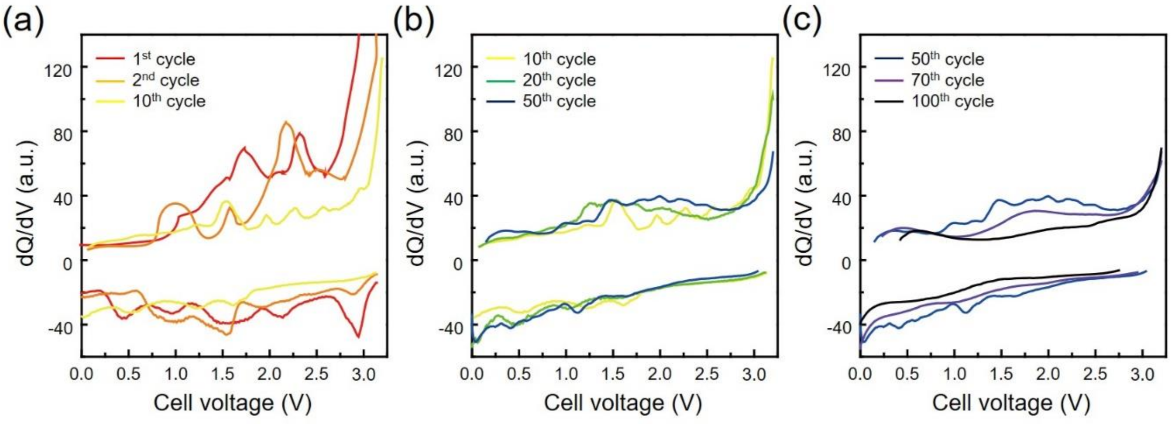

Figure S35. dQ/dV plots derived from the representative cycles of the all-organic cell based on DMQA. Faradaic peaks are diminished as cycles proceeded, which is mainly attributed to the degradation of the anode side.

6. Redox mechanism of DMQA during anion- and lithium- coupled reactions

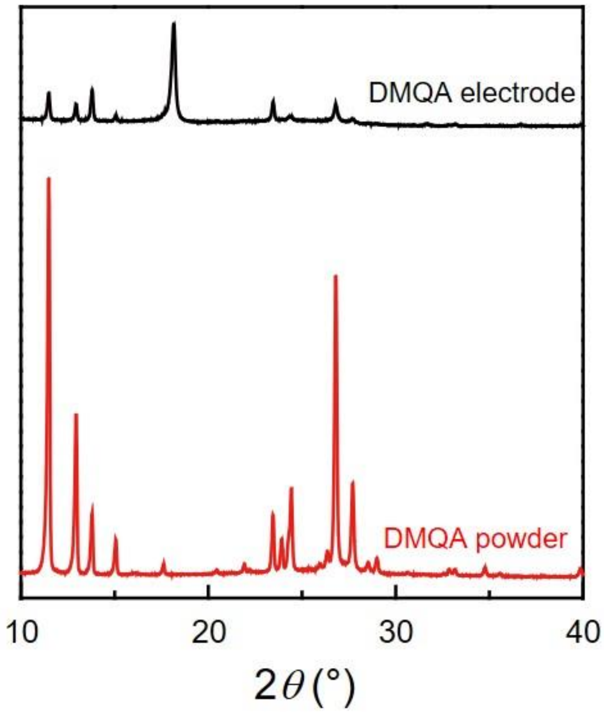

Figure S36. XRD patterns of the (red) DMQA powder and (black) DMQA electrode.

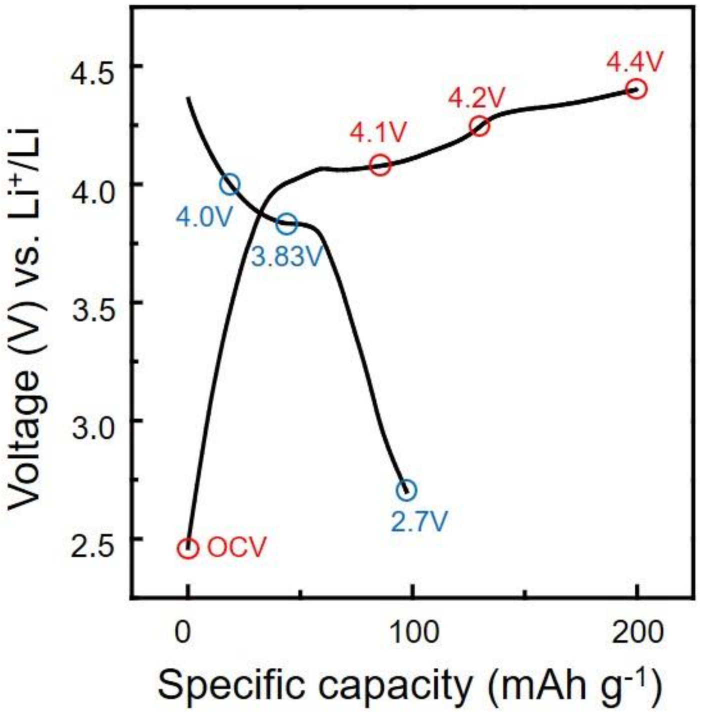

Figure S37. The voltage profile with sample harvesting stages for the ex-situ XRD analysis.

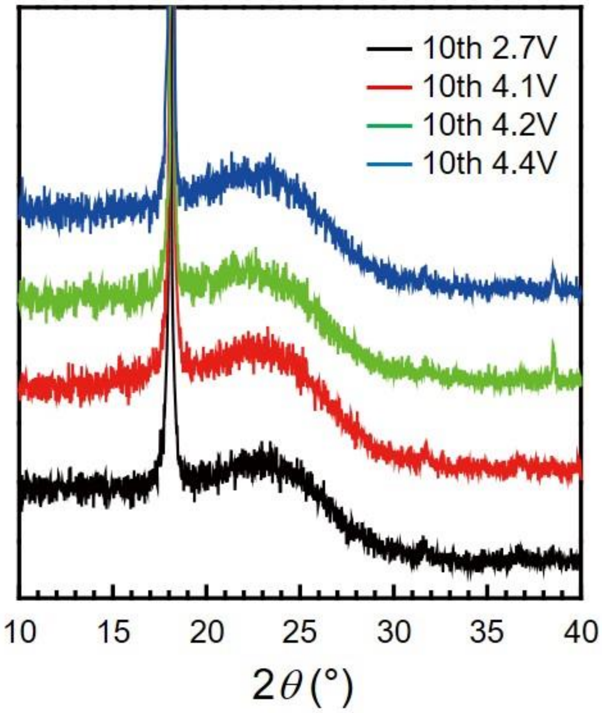

Figure S38. XRD patterns from the cells harvested from the different states at the 10th cycle

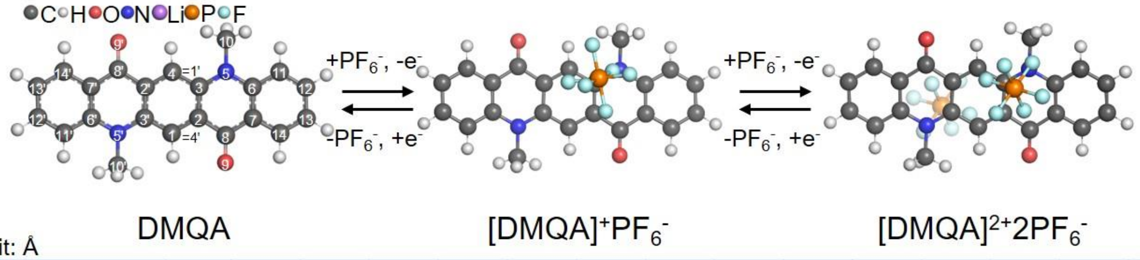

Unit: Å

	C1-C2	C2-C3	C3-C4	C3-N5	N5-C6	C6-C7	C7-C8	C8-C2	N5-C10	C8=O9	C6-C11	C11-C12	C12-C13	C13-C14	C14-C7
**DMQA**	1.394	1.420	1.398	1.392	1.387	1.420	1.462	1.474	1.467	1.238	1.415	1.382	1.404	1.379	1.409
**$[DMQA]^{+}PF_6^{-}$**	1.377	1.428	1.415	1.366	1.403	1.416	1.468	1.481	1.476	1.227	1.408	1.386	1.399	1.386	1.400
**$[DMQA]^{+}2PF_6^{-}$**	1.358	1.442	1.438	1.338	1.413	1.415	1.469	1.490	1.485	1.219	1.406	1.389	1.394	1.393	1.391

**$[DMQA]^{+}PF_6^{-}$ – DMQA**	-0.017	0.008	0.017	-0.026	0.016	-0.004	0.006	0.007	0.009	-0.011	-0.007	0.004	-0.005	0.007	-0.009
**$[DMQA]^{+}2PF_6^{-}$ – DMQA**	-0.036	0.022	0.040	-0.054	0.026	-0.005	0.007	0.016	0.018	-0.019	-0.009	0.007	-0.01	0.014	-0.018

Figure S39. Bond length alterations of DMQA during oxidation accompanied by anion-association. The upper table displays absolute values for the major bond lengths and the lower table tabulates their relative change during oxidation of DMQA. The most significant bond length alteration occurs at amine groups (C3-N5). The major alteration is accompanied with minor bond length alterations in order to effectively distribute charge through the conjugated carbon ring of the DMQA molecule (C1-C2, C2-C3, C3-C4). The major oxidation centers are amine groups while the conjugated carbon chain also plays the supportive role in charge withdrawal to prevent huge local deformation in the structure of DMQA molecule.

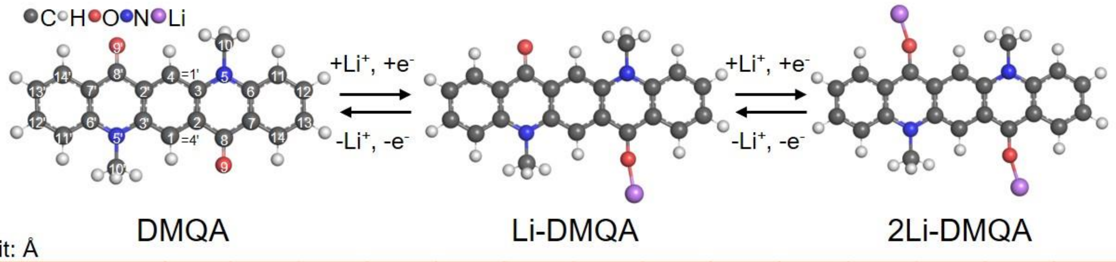

Unit: Å

	C1-C2	C2-C3	C3-C4	C3-N5	N5-C6	C6-C7	C7-C8	C8-C2	N5-C10	C8=O9	C6-C11	C11-C12	C12-C13	C13-C14	C14-C7
**DMQA**	1.394	1.420	1.398	1.392	1.387	1.420	1.462	1.474	1.467	1.238	1.415	1.382	1.404	1.379	1.409
**Li-DMQA (right side)**	1.418	1.432	1.388	1.398	1.391	1.423	1.454	1.428	1.462	1.281	1.409	1.391	1.399	1.386	1.409
**Li-DMQA (left side)**	1.418	1.427	1.392	1.398	1.391	1.420	1.468	1.442	1.462	1.259	1.412	1.389	1.400	1.386	1.406
**2Li-DMQA**	1.442	1.443	1.378	1.406	1.395	1.425	1.458	1.400	1.457	1.306	1.405	1.398	1.394	1.393	1.407

Right side: a half-side which contains lithium
Left side: a half-side which does not contain lithium

**Li-DMQA (right) – DMQA**	0.024	0.012	-0.01	0.006	0.004	0.003	-0.008	-0.046	-0.005	0.043	-0.006	0.009	-0.005	0.007	0.000
**Li-DMQA (left) – DMQA**	0.024	0.007	-0.006	0.006	0.004	0.000	0.006	-0.032	-0.005	0.021	-0.003	0.007	-0.004	0.007	-0.003
**2Li-DMQA – DMQA**	0.048	0.023	-0.02	0.014	0.008	0.005	-0.004	-0.074	-0.01	0.068	-0.01	0.016	-0.01	0.014	-0.002

Figure S40. Bond length alterations of DMQA during lithium-coupled reduction. Absolute values for the major bond lengths are tabulated in the upper table. The lower table shows the relative change of bond lengths. Significant increase of bond lengths in C1-C2, and C8=O9 and decrease of bond lengths of C2-C8 and as well as small changes in bond lengths among other carbon atoms in the central aromatic ring are observed. That suggests carbonyl oxygen groups as major redox centers and the supportive role of the central aromatic ring in charge storage mechanism.

7. References

[1] M. Lee, J. Hong, B. Lee, K. Ku, S. Lee, C. B. Park, K. Kang, *Green Chem.* **2017**, *19*, 2980-2985.

[2] M. J. Frisch, G. W. Trucks, H. B. Schlegel, G. E. Scuseria, M. A. Robb, J. R. Cheeseman, G. Scalmani, V. Barone, G. A. Petersson, H. Nakatsuji, X. Li, M. Caricato, A. V. Marenich, J. Bloino, B. G. Janesko, R. Gomperts, B. Mennucci, H. P. Hratchian, J. V. Ortiz, A. F. Izmaylov, J. L. Sonnenberg, Williams, F. Ding, F. Lipparini, F. Egidi, J. Goings, B. Peng, A. Petrone, T. Henderson, D. Ranasinghe, V. G. Zakrzewski, J. Gao, N. Rega, G. Zheng, W. Liang, M. Hada, M. Ehara, K. Toyota, R. Fukuda, J. Hasegawa, M. Ishida, T. Nakajima, Y. Honda, O. Kitao, H. Nakai, T. Vreven, K. Throssell, J. A. Montgomery Jr., J. E. Peralta, F. Ogliaro, M. J. Bearpark, J. J. Heyd, E. N. Brothers, K. N. Kudin, V. N. Staroverov, T. A. Keith, R. Kobayashi, J. Normand, K. Raghavachari, A. P. Rendell, J. C. Burant, S. S. Iyengar, J. Tomasi, M. Cossi, J. M. Millam, M. Klene, C. Adamo, R. Cammi, J. W. Ochterski, R. L. Martin, K. Morokuma, O. Farkas, J. B. Foresman, D. J. Fox, Gaussian Inc., Wallingford, CT, **2016**.

[3] a) A. D. Becke, *J. Chem. Phys.* **1993**, *98*, 5648-5652; b) P. J. Stephens, F. J. Devlin, C. F. Chabalowski, M. J. Frisch, *J. Phys. Chem.* **1994**, *98*, 11623-11627.

[4] J. Tomasi, B. Mennucci, R. Cammi, *Chem. Rev.* **2005**, *105*, 2999-3094.

[5] Schrodinger, LLC. The PyMOL Molecular Graphics System, Version 1.8. **2015**.

[6] I. A. Rodríguez-Pérez, Z. Jian, P. K. Waldenmaier, J. W. Palmisano, R. S. Chandrabose, X. Wang, M. M. Lerner, R. G. Carter, X. Ji, *ACS Energy Lett.* **2016**, *1*, 719-723.

[7] Y. Inatomi, N. Hojo, T. Yamamoto, S.-i. Watanabe, Y. Misaki, *ChemPlusChem* **2012**, *77*, 973-976.

[8] S. Nishida, Y. Yamamoto, T. Takui, Y. Morita, *ChemSusChem* **2013**, *6*, 794-797.

[9] T. Yokoji, H. Matsubara, M. Satoh, *J. Mater. Chem. A* **2014**, *2*, 19347-19354.

[10] T. Yokoji, Y. Kameyama, N. Maruyama, H. Matsubara, *J. Mater. Chem. A* **2016**, *4*, 5457-5466.

[11] S. Gottis, A.-L. Barrès, F. Dolhem, P. Poizot, *ACS Appl. Mater. Interfaces* **2014**, *6*, 10870-10876.

[12] Y. Liang, P. Zhang, J. Chen, *Chem. Sci.* **2013**, *4*, 1330-1337.

[13] Y. Liang, P. Zhang, S. Yang, Z. Tao, J. Chen, *Adv. Energy Mater.* **2013**, *3*, 600-605.

[14] M. Yao, H. Senoh, S.-i. Yamazaki, Z. Siroma, T. Sakai, K. Yasuda, *J. Power Sources* **2010**, *195*, 8336-8340.

[15] M. Lee, J. Hong, D.-H. Seo, D. H. Nam, K. T. Nam, K. Kang, C. B. Park, *Angew. Chem. Int. Ed.* **2013**, *52*, 8322-8328.

[16] R. Zeng, L. Xing, Y. Qiu, Y. Wang, W. Huang, W. Li, S. Yang, *Electrochim. Acta* **2014**, *146*, 447-454.

[17] S. Wang, L. Wang, K. Zhang, Z. Zhu, Z. Tao, J. Chen, *Nano Lett.* **2013**, *13*, 4404-4409.

[18] Z. Luo, L. Liu, Q. Zhao, F. Li, J. Chen, *Angew. Chem. Int. Ed.* **2017**, *56*, 12561-12565.

[19] T. Yokoji, Y. Kameyama, S. Sakaida, N. Maruyama, M. Satoh, H. Matsubara, *Chem. Lett.* **2015**, *44*, 1726-1728.

[20] J. Xie, W. Chen, Z. Wang, K. C. W. Jie, M. Liu, Q. Zhang, *Chem. Asian J.* **2017**, *12*, 868-876.

[21] W. Wan, H. Lee, X. Yu, C. Wang, K.-W. Nam, X.-Q. Yang, H. Zhou, *RSC Adv.* **2014**, *4*, 19878-19882.

[22] A. Shimizu, H. Kuramoto, Y. Tsujii, T. Nokami, Y. Inatomi, N. Hojo, H. Suzuki, J.-i. Yoshida, *J. Power Sources* **2014**, *260*, 211-217.

[23] M. Yao, S.-i. Yamazaki, H. Senoh, T. Sakai, T. Kiyobayashi, *Mater. Sci. Eng. B* **2012**, *177*, 483-487.

[24] B. Tian, Z. Ding, G.-H. Ning, W. Tang, C. Peng, B. Liu, J. Su, C. Su, K. P. Loh, *Chem. Commun.* **2017**, *53*, 2914-2917.

[25] C. Luo, R. Huang, R. Kevorkyants, M. Pavanello, H. He, C. Wang, *Nano Lett.* **2014**, *14*, 1596-1602.

[26] L. Fédèle, F. Sauvage, J. Bois, J.-M. Tarascon, M. Bécuwe, *J. Electrochem. Soc.* **2013**, *161*, A46-A52.

[27] M. Armand, S. Grugeon, H. Vezin, S. Laruelle, P. Ribière, P. Poizot, J. M. Tarascon, *Nat. Mater.* **2009**, *8*, 120-125.

[28] L. Wang, C. Mou, B. Wu, J. Xue, J. Li, *Electrochim. Acta* **2016**, *196*, 118-124.

[29] L. Fédèle, F. Sauvage, S. Gottis, C. Davoisne, E. Salager, J.-N. Chotard, M. Becuwe, *Chem. Mater.* **2017**, *29*, 546-554.

[30] A. S. Pavitt, E. J. Bylaska, P. G. Tratnyek, *Environ. Sci.: Process. Impacts* **2017**, *19*, 339-349.

[31] a) K. N. Wood, G. Teeter, *ACS Appl. Energy Mater.* **2018**, *1*, 4493-4504; b) R. M. Kasse, N. R. Geise, J. S. Ko, J. Nelson Weker, H.-G. Steinrück, M. F. Toney, *J. Mater. Chem. A* **2020**, *8*, 16960-16972.

[32] S. Jiao, X. Ren, R. Cao, M. H. Engelhard, Y. Liu, D. Hu, D. Mei, J. Zheng, W. Zhao, Q. Li, N. Liu, B. D. Adams, C. Ma, J. Liu, J.-G. Zhang, W. Xu, *Nat. Energy* **2018**, *3*, 739-746.

[33] a) T. Kondo, D. Guo, T. Shikano, T. Suzuki, M. Sakurai, S. Okada, J. Nakamura, *Sci. Rep.* **2015**, *5*, 16412; b) I. Bertóti, M. Mohai, K. László, *Carbon* **2015**, *84*, 185-196
